# Supplementary material for: Arabidopsis TRB proteins function in H3K4me3 demethylation by recruiting JMJ14
Source: Nat Commun. 2023 Mar 28;14:1736. doi: 10.1038/s41467-023-37263-9 (PMC10049986; doi:10.1038/s41467-023-37263-9)
Supplement: Supplementary file 1 — Supplementary Information [file 41467_2023_37263_MOESM1_ESM.pdf]

Supplementary Information

Arabidopsis TRB proteins function in H3K4me3 demethylation by recruiting JMJ14.

Ming Wang[1]*, Zhenhui Zhong[1]*, Javier Gallego-Bartolomé[1,7], Suhua Feng[1,2], Yuan-Hsin Shih[1,3], Mukun Liu[1], Jessica Zhou[1], John Curtis Richey[1], Charmaine Ng[1], Yasaman Jami-Alahmadi[4], James Wohlschlegel[4], Keqiang Wu[3], and Steven E. Jacobsen[1,2,4,5,6].

[1]Department of Molecular, Cell and Developmental Biology, University of California at Los Angeles, Los Angeles, CA 90095, USA

[2]Eli & Edythe Broad Center of Regenerative Medicine & Stem Cell Research, University of California at Los Angeles, Los Angeles, CA 90095, USA

[3]Institute of Plant Biology, National Taiwan University, Taipei, 10617 Taiwan

[4]Department of Biological Chemistry, University of California at Los Angeles, CA 90095, USA

[5]Howard Hughes Medical Institute (HHMI), University of California at Los Angeles, Los Angeles, CA 90095, USA

[6]Correspondence: jacobsen@ucla.edu

[7]Present address: Instituto de Biología Molecular y Celular de Plantas (IBMCP), CSIC-Universitat Politècnica de València, 46022 Valencia, Spain

*These authors contributed equally

Supplementary Figs. 1-18

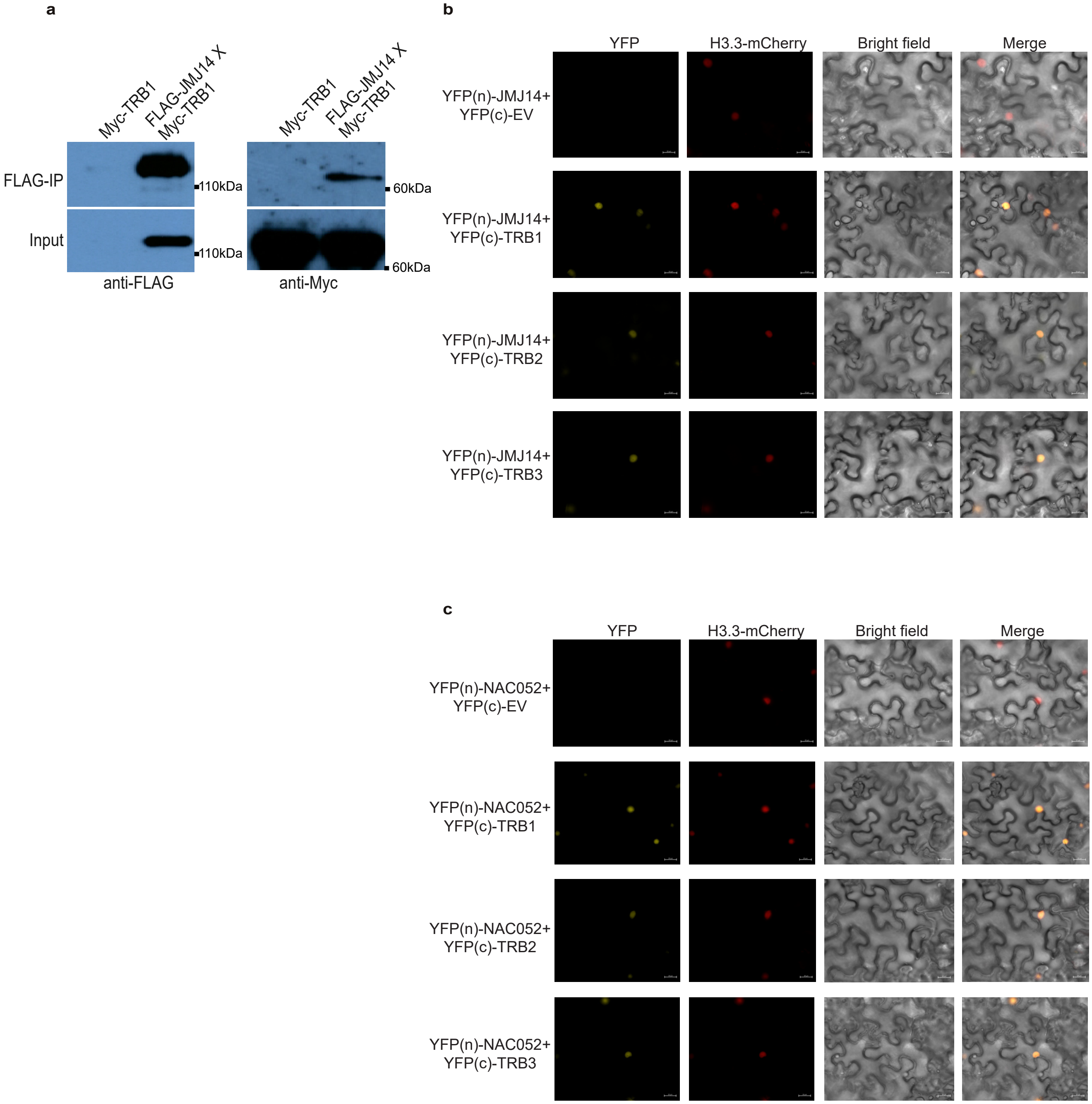

Supplementary Fig. 1 FLAG-TRB1 interacts with Myc-JMJ14. a Western blot showing the Co-immunoprecipitation (Co-IP) assay in FLAG-JMJ14 and Myc-TRB1 F2 crossed lines. **b-c** BiFC assays showing the YFP signals of leaves transiently co-expressing cYFP-TRBs and nYFP-JMJ14 (**b**) or nYFP-NAC052 (**c**). All the scale bars are 20 μm in length, and similar results were obtained in two independent replicates (**a-c**).

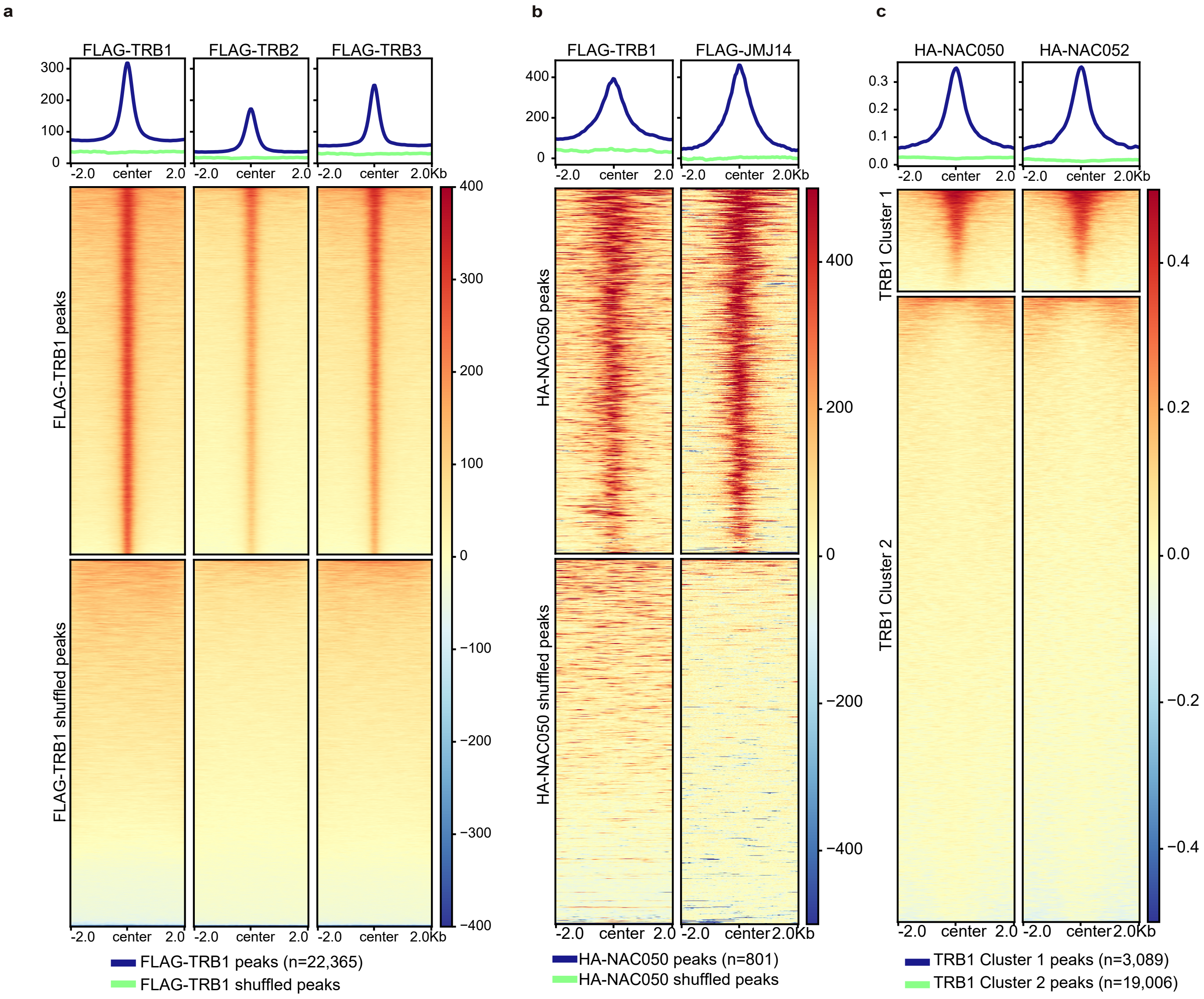

Supplementary Fig. 2 TRBs are partially colocalized with JMJ14, NAC050, and NAC052. a-c Metaplots and heatmaps showing FLAG-TRB1, 2, 3 ChIP-seq signals over FLAG-TRB1 peaks and shuffled peaks (**a**, n=22,365), FLAG-TRB1 and FLAG-JMJ14 ChIP-seq signals over HA-NAC050 peaks (n=801) and shuffled peaks (**b**), and HA-NAC050 and HA-NAC052 ChIP-seq signals over the TRB1 Cluster 1 peaks (n=3,089) and the Cluster 2 peaks (n=19,006) (**c**).

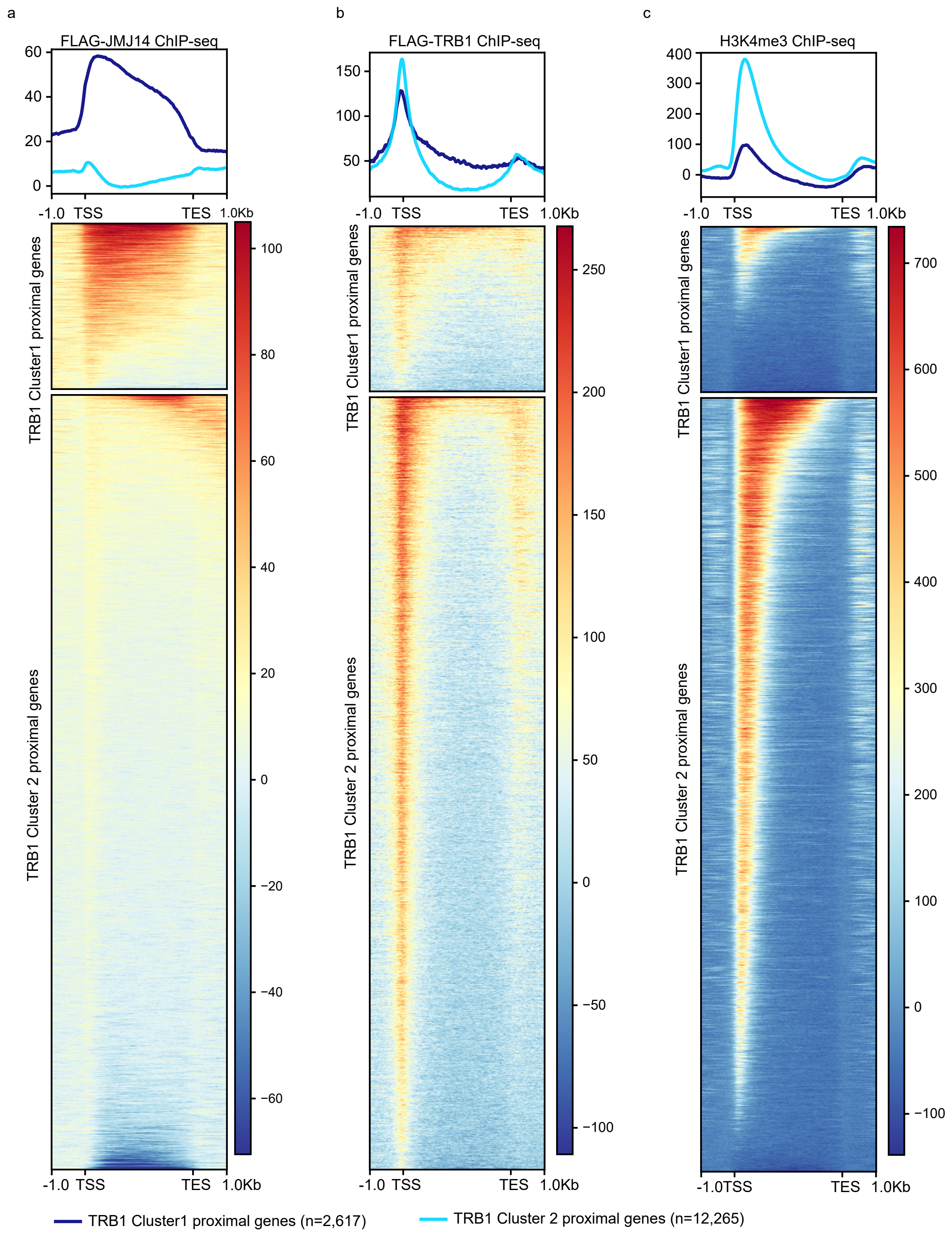

Supplementary Fig. 3 JMJ14 and TRB1 are colocalized at gene body regions. a-c Heatmaps and metaplots representing FLAG-JMJ14 (**a**), FLAG-TRB1 (**b**), and H3K4me3 (**c**) ChIP-seq signals over the TRB1 Cluster 1 proximal genes (n=2,617) and the Cluster 2 proximal genes (n=12,265).

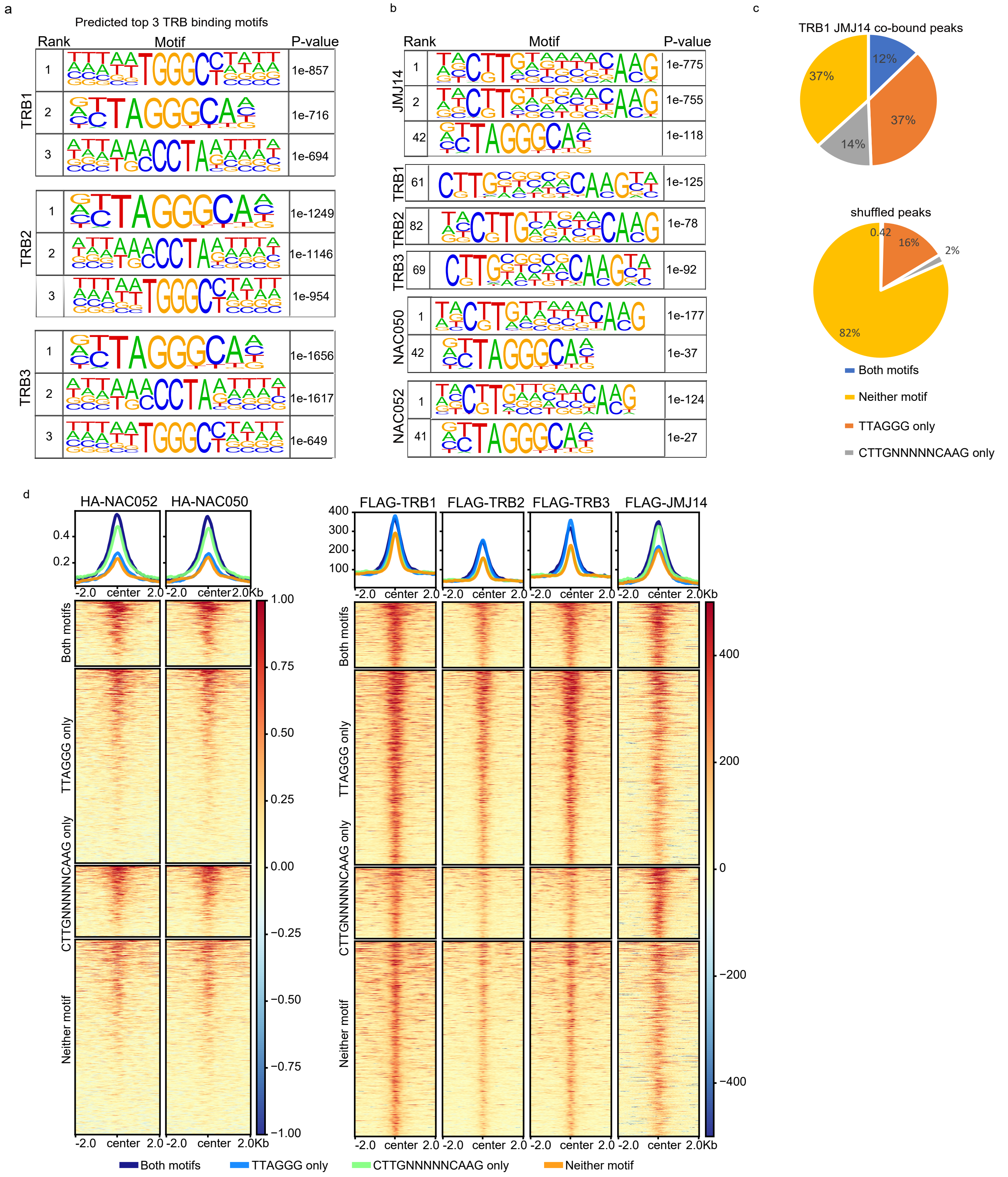

Supplementary Fig. 4 Predicted TRB binding motifs include the telomeric repeat DNA sequence TTAGGG and the known JMJ14 binding motif CTTGnnnnnCAAG. a Motif prediction by Homer showing the top 3 binding motifs of TRB1, TRB2, and TRB3. **b** Motif prediction by Homer showing that JMJ14, TRBs, NAC050, and NAC052 are all predicted to bind the TTAGGG and CTTGnnnnnCAAG motifs. **c** Pie charts indicate the percentile of the TRB1 and JMJ14 co-bound peaks (top panel) and shuffled peaks (bottom panel) displaying the TTAGGG motif only (n=1,176), the CTTGnnnnnCAAG motif only (n=431), both motifs (n=396), and neither (n=1,180), respectively. **d** Heatmaps and metaplots representing the normalized HA-NAC050/052, FLAG-TRB1, FLAG-TRB2, FLAG-TRB3, and FLAG-JMJ14 ChIP-seq signals over TRB1 and JMJ14 co-bound regions displaying the TTAGGG motif only, the CTTGnnnnnCAAG motif only, both motifs, and neither, respectively.

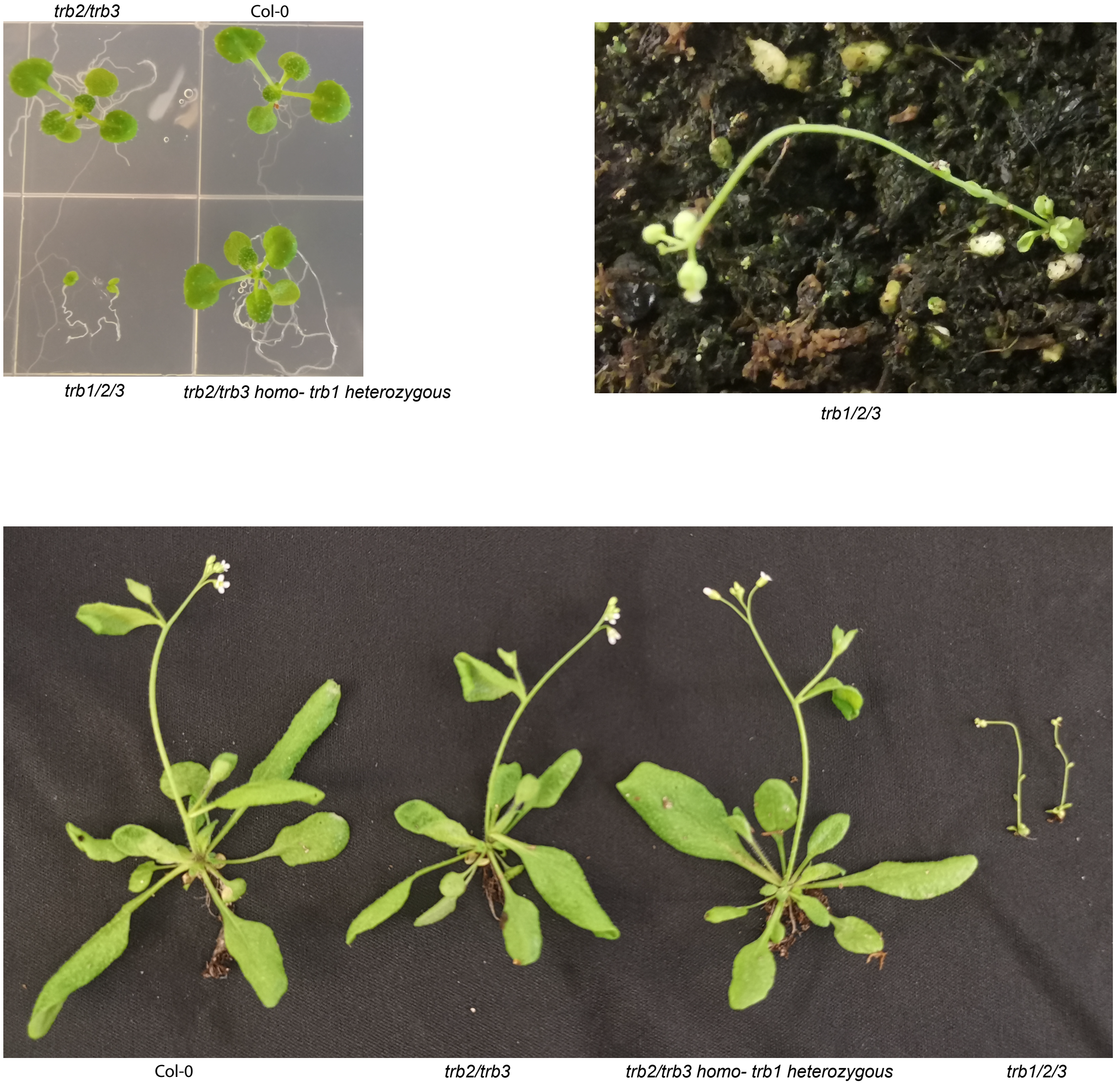

Supplementary Fig. 5 The *trb1/2/3* triple mutant shows strong morphological defects. The photos show the phenotype of *trb2/3* double mutant, Col-0, *trb1/2/3* triple mutant, and *trb2/3* homo- *trb1* heterozygous mutant grown on MS medium for 2-3 weeks (top left), and then transferred to soil for another 2 weeks (bottom). The top right photo shows a closer view of a *trb1/2/3* triple mutant on soil.

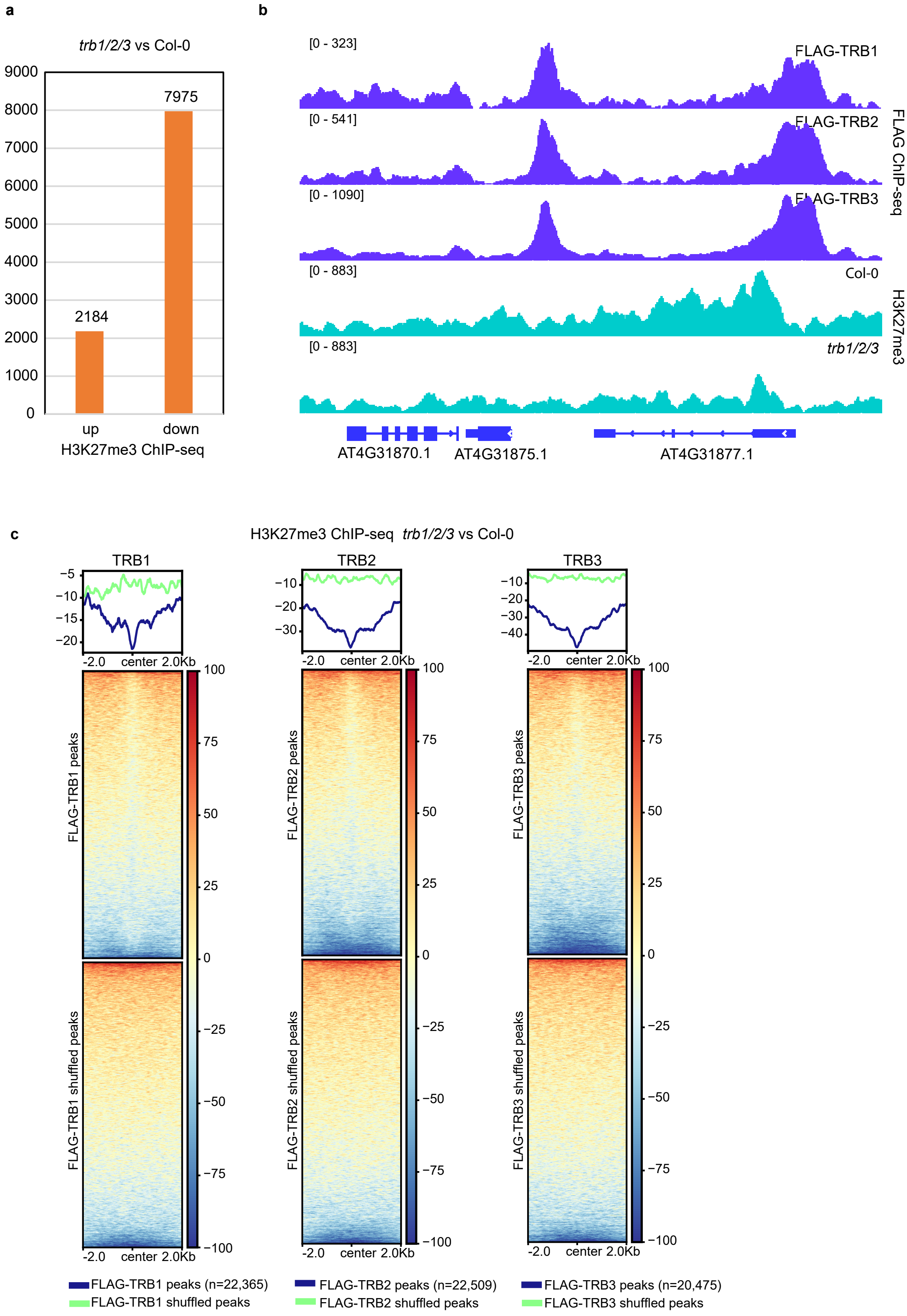

Supplementary Fig. 6 H3K27me3 ChIP-seq signals are reduced in the *trb1/2/3* mutant. a Bar chart indicates the number of regions with up- or down-regulated H3K27me3 ChIP-seq signals in the *trb1/2/3* mutant, respectively. **b** Screenshots showing FLAG ChIP-seq signals in FLAG-TRB1, FLAG-TRB2, and FLAG-TRB3 (top three lanes), and H3K27me3 ChIP-seq signals in Col-0 and the *trb1/2/3* mutant, over a representative region. **c** Metaplots and heatmaps representing H3K27me3 ChIP-seq signals in the *trb1/2/3* mutant versus Col-0 wild type over FLAG-TRB1 peaks and shuffled peaks (n=22,365, left panel), FLAG-TRB2 peaks and shuffled peaks (n=22,509, middle panel), and FLAG-TRB3 peaks and shuffled peaks (n=20,475, right panel).

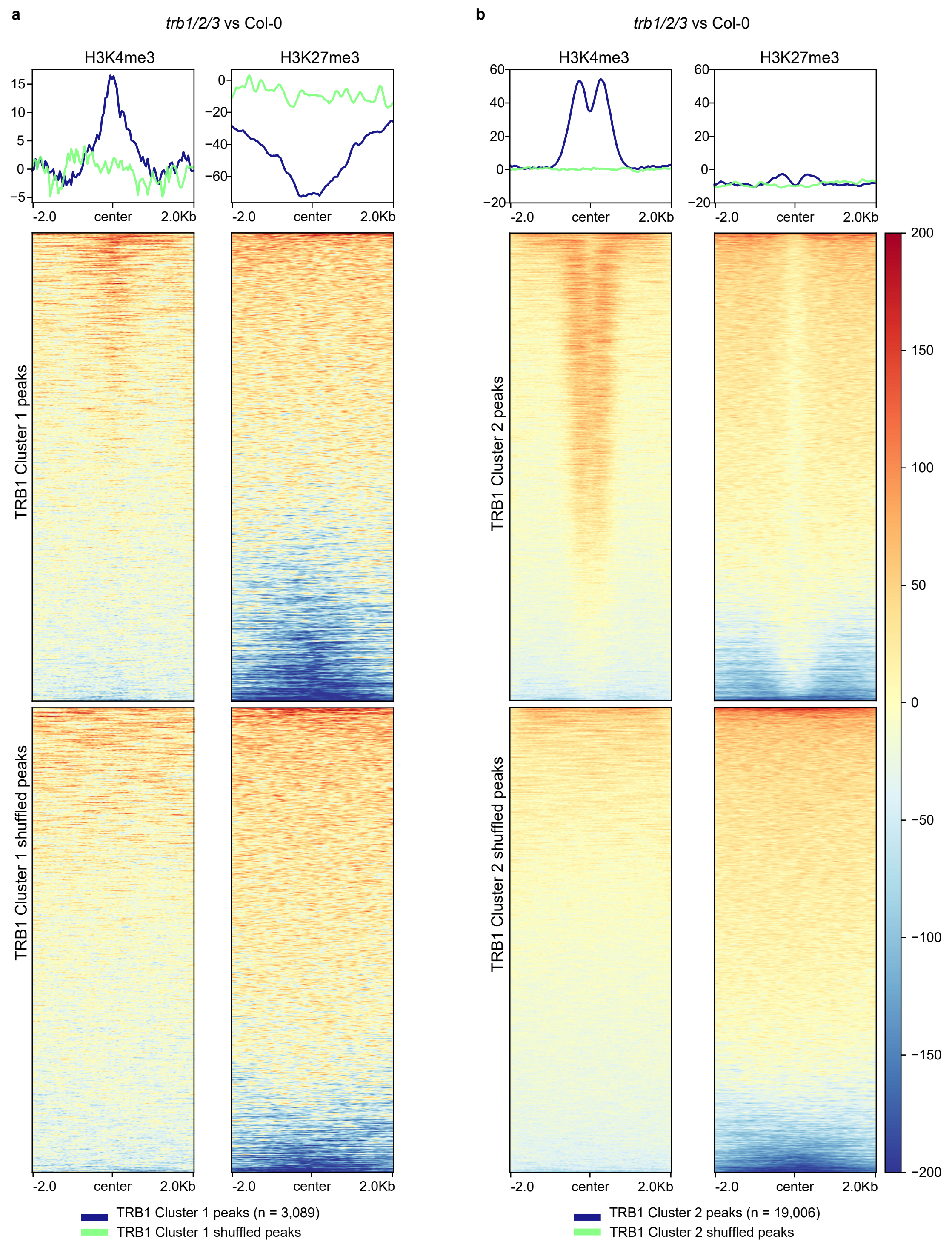

Supplementary Fig. 7 Changes of H3K4me3 and H3K27me3 ChIP-seq signals in the *trb1/2/3* mutant displayed opposite trends at TRB1-JMJ14 co-bound regions. a-b Metaplots and heatmaps depicting the normalized H3K4me3 and H3K27me3 ChIP-seq signals in the *trb1/2/3* mutant versus Col-0 at TRB1 Cluster 1 peaks and shuffled peaks (**a**, n=3,089), and TRB1 Cluster 2 peaks and shuffled peaks (**b**, n=19,006), respectively.

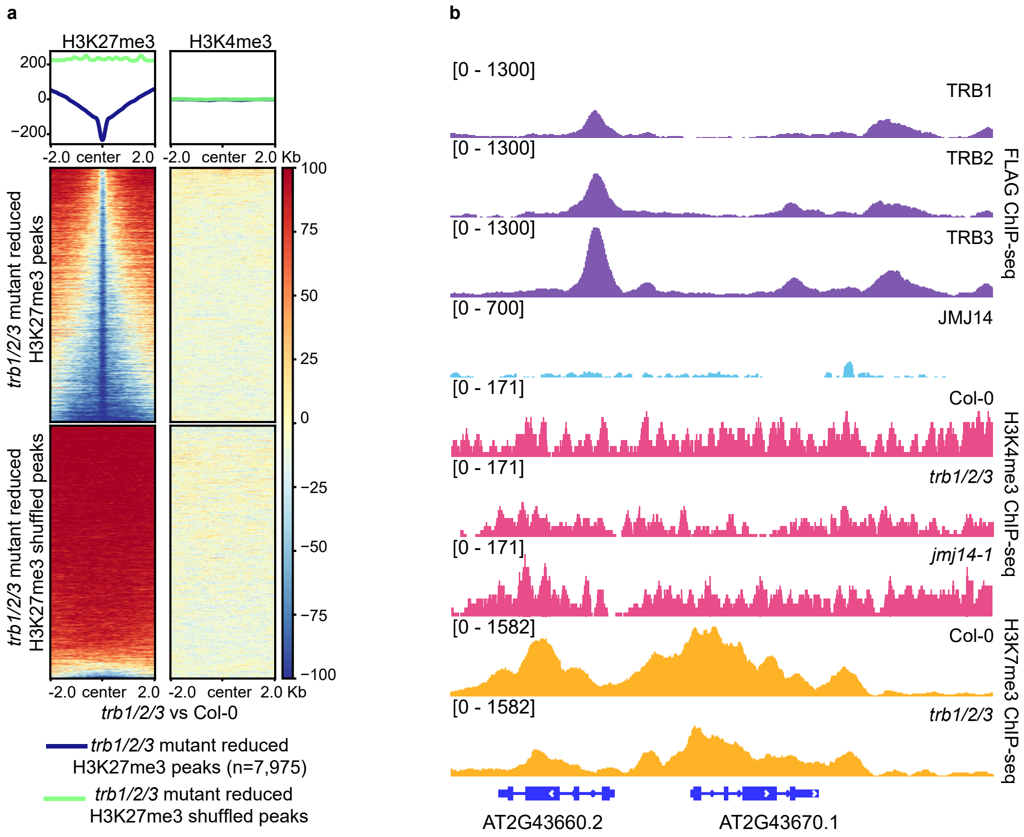

Supplementary Fig. 8 The reduction of H3K27me3 does not always trigger an increase of H3K4me3 in the *trb1/2/3* mutant. a Metaplots and heatmaps depicting H3K27me3 and H3K4me3 ChIP-seq signals over the reduced H3K27me3 peaks and shuffled peaks (n=7,975) in the *trb1/2/3* mutants versus Col-0 wild type. **b** Screenshots showing FLAG ChIP-seq signals of FLAG-TRB1, FLAG-TRB2, FLAG-TRB3, and FLAG-JMJ14, H3K4me3 ChIP-seq signals in Col-0, *trb1/2/3,* and *jmj14-1* mutants, and H3K27me3 ChIP-seq signals in Col-0 and the *trb1/2/3* mutant over a representative region.

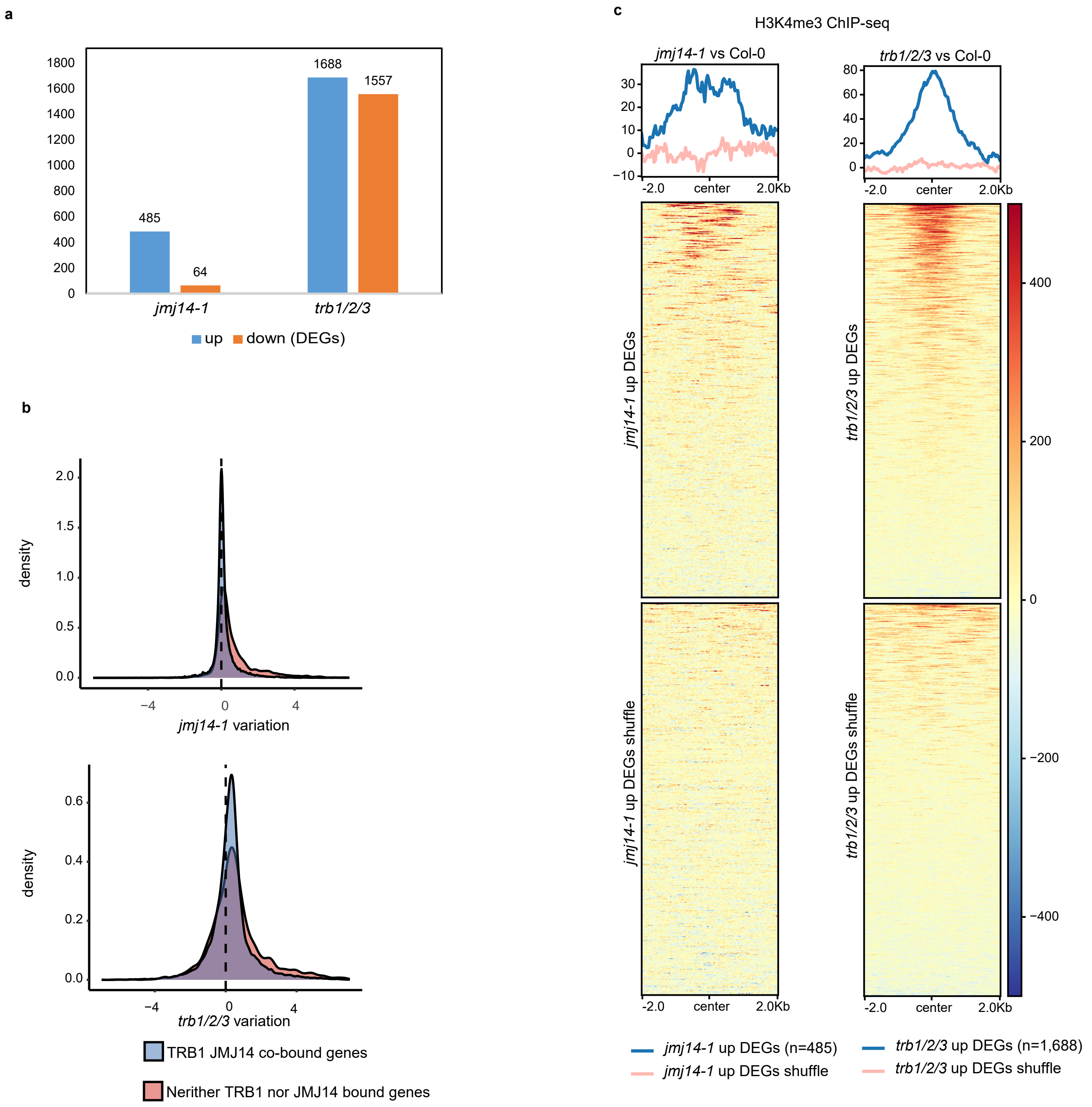

Supplementary Fig. 9 *trb1/2/3* and *jmj14-1* up-regulated DEGs are associated with H3K4me3 induction. a Bar chart indicates the number of up- and down-regulated DEGs in *jmj14-1* and *trb1/2/3* mutants, respectively. **b** Density plots of DEGs in *jmj14-1* (upper panel) and *trb1/2/3* (lower panel) mutants over the TRB1 and JMJ14 co-bound genes and neither TRB1 nor JMJ14 bound genes, respectively. **c** Metaplots and heatmaps representing the normalized H3K4me3 ChIP-seq signals in *jmj14-1* mutant versus Col-0 over *jmj14-1* mutant up-regulated DEGs or shuffle sites (n=485, left panel), and in *trb1/2/3* mutants versus Col-0 over *trb1/2/3* mutant up-regulated DEGs or shuffle sites (n=1,688, right panel).

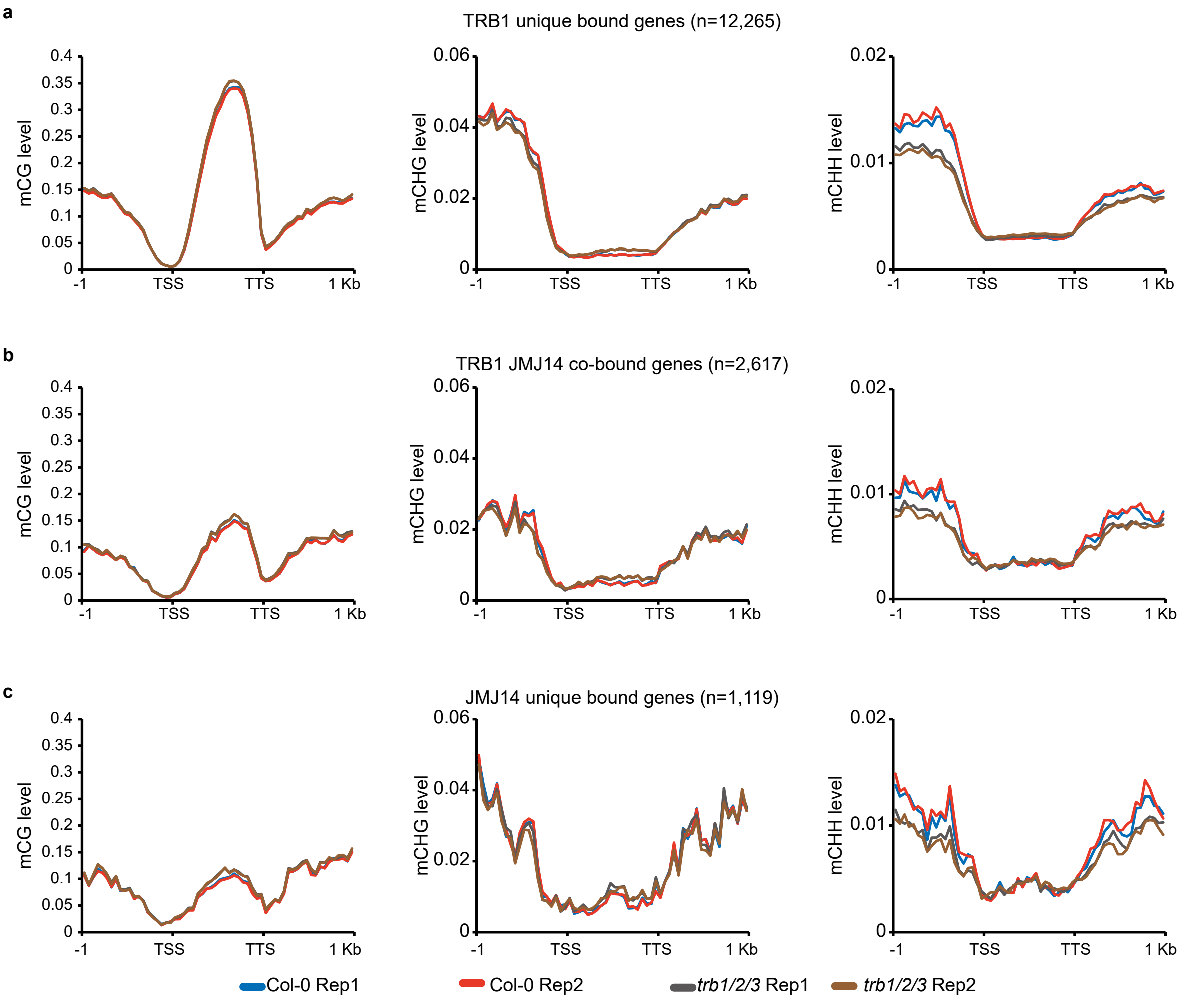

Supplementary Fig. 10 CHH DNA methylation level is reduced in the *trb1/2/3* mutant. a-c CG, CHG, and CHH DNA methylation levels in two replicates of Col-0 and *trb1/2/3* triple mutants over 1 kb flanks of TRB1 unique bound genes (**a**, n=12,265), TRB1-JMJ14 co-bound genes (**b**, n=2,617), and JMJ14 unique bound genes (**c**, n=1,119). The DNA methylation levels were measured by whole genome bisulfite sequencing.

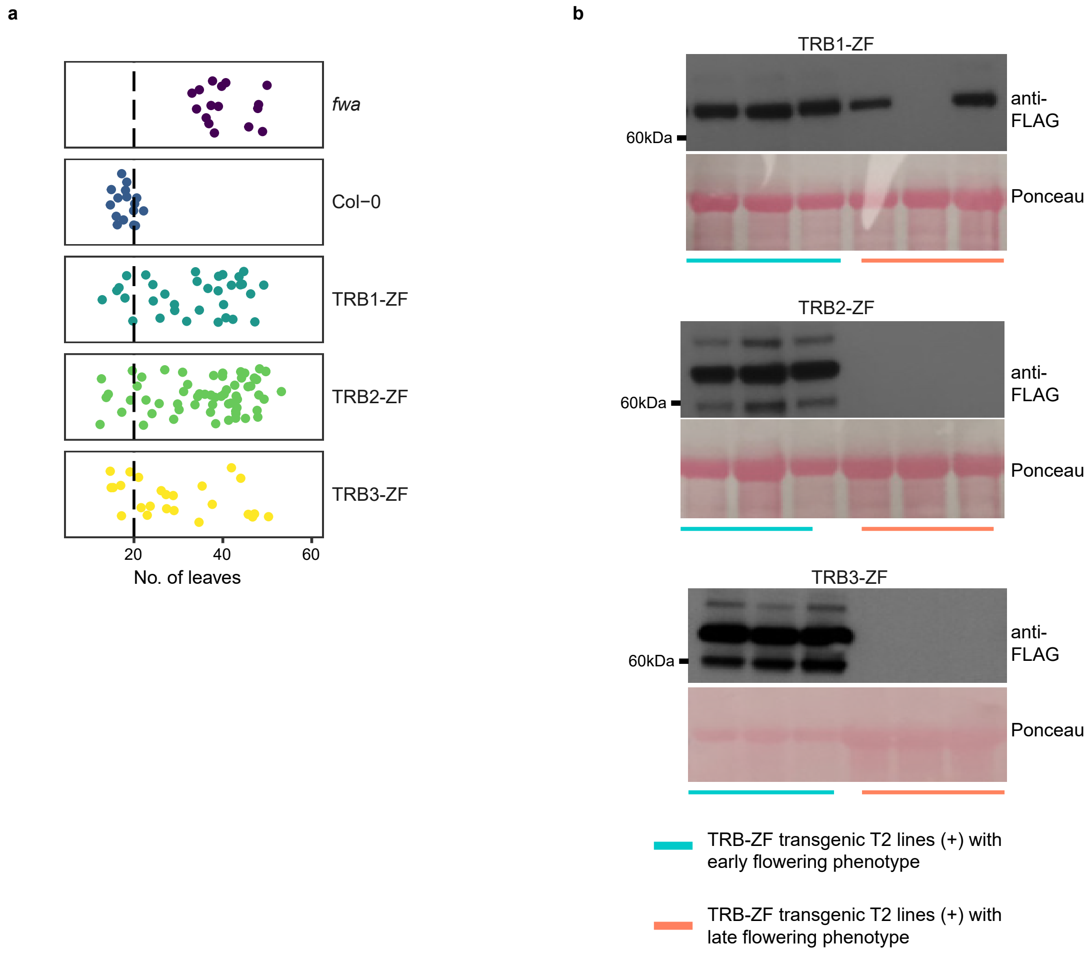

Supplementary Fig. 11 TRB-ZF T1 lines restore an early flowering phenotype in *fwa*. a Dot plots showing the leaf count of *fwa*, Col-0, and the T1 lines of TRB1-ZF, TRB2-ZF, and TRB3-ZF. **b** Western blot of the TRB-ZF fusions in T2 transgenic lines showing early flowering phenotypes (left three samples) and late flowering phenotypes (right three samples), respectively. Similar results were observed in two biological replicates.

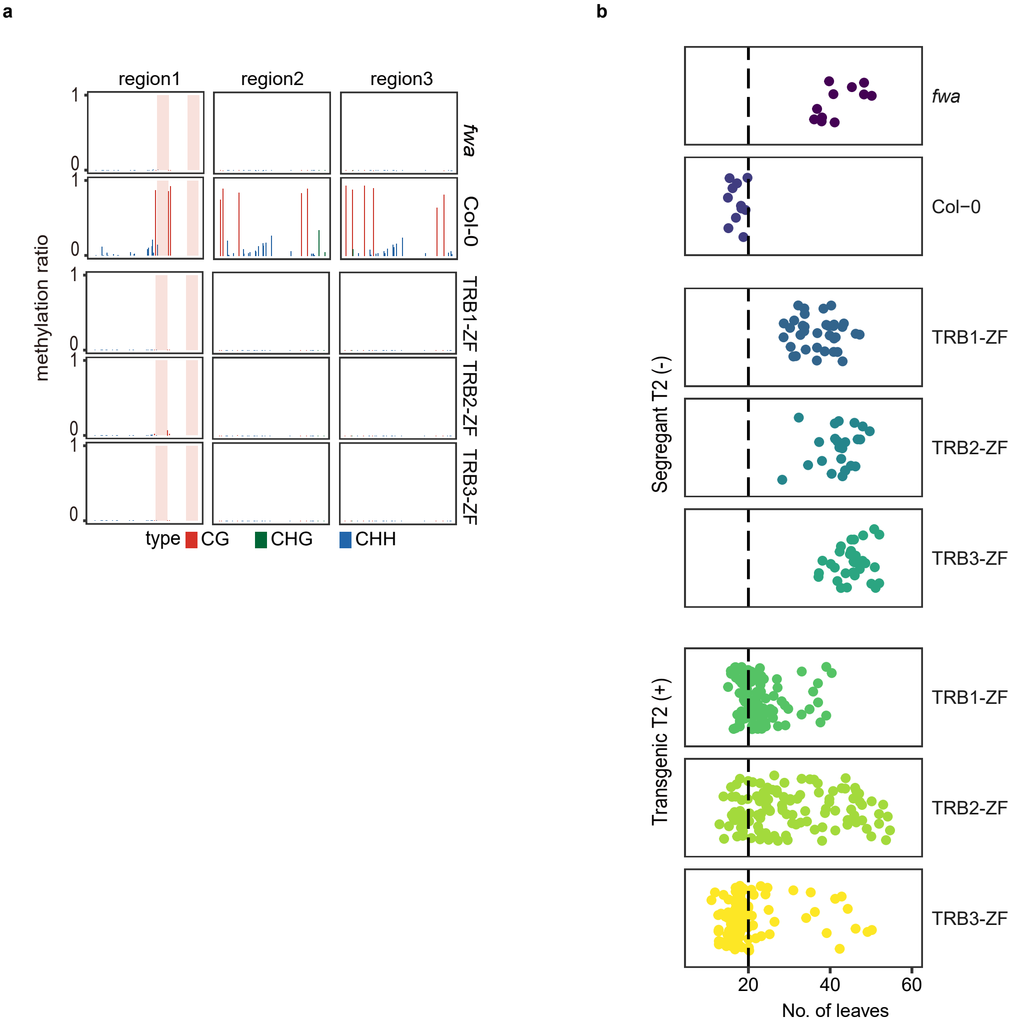

Supplementary Fig. 12 The TRB-ZF fusions triggered early flowering phenotype is not associated with DNA methylation and is not heritable in T2 null segregant lines. a CG, CHG, and CHH DNA methylation level over *FWA* promoter regions measured by bisulfite PCR-seq. Pink vertical boxes indicate ZF binding sites. **b** Dot plots represent the leaf counts of *fwa*, Col-0, null segregant and transgenic T2 lines of TRB1-ZF, TRB2-ZF, and TRB3-ZF, respectively.

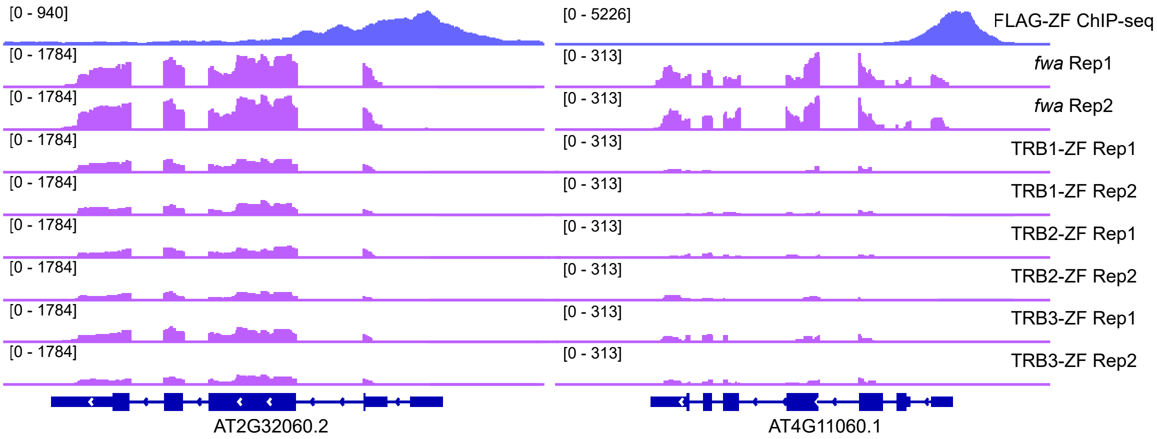

Supplementary Fig. 13 TRB-ZF fusions triggered gene silencing over two representative ZF off-target sites. Screenshots showing two replicates of RNA-seq signals in *fwa*, TRB1-ZF, TRB2-ZF, and TRB3-ZF over two representative ZF off target sites. FLAG-ZF ChIP-seq indicates the ZF binding sites.

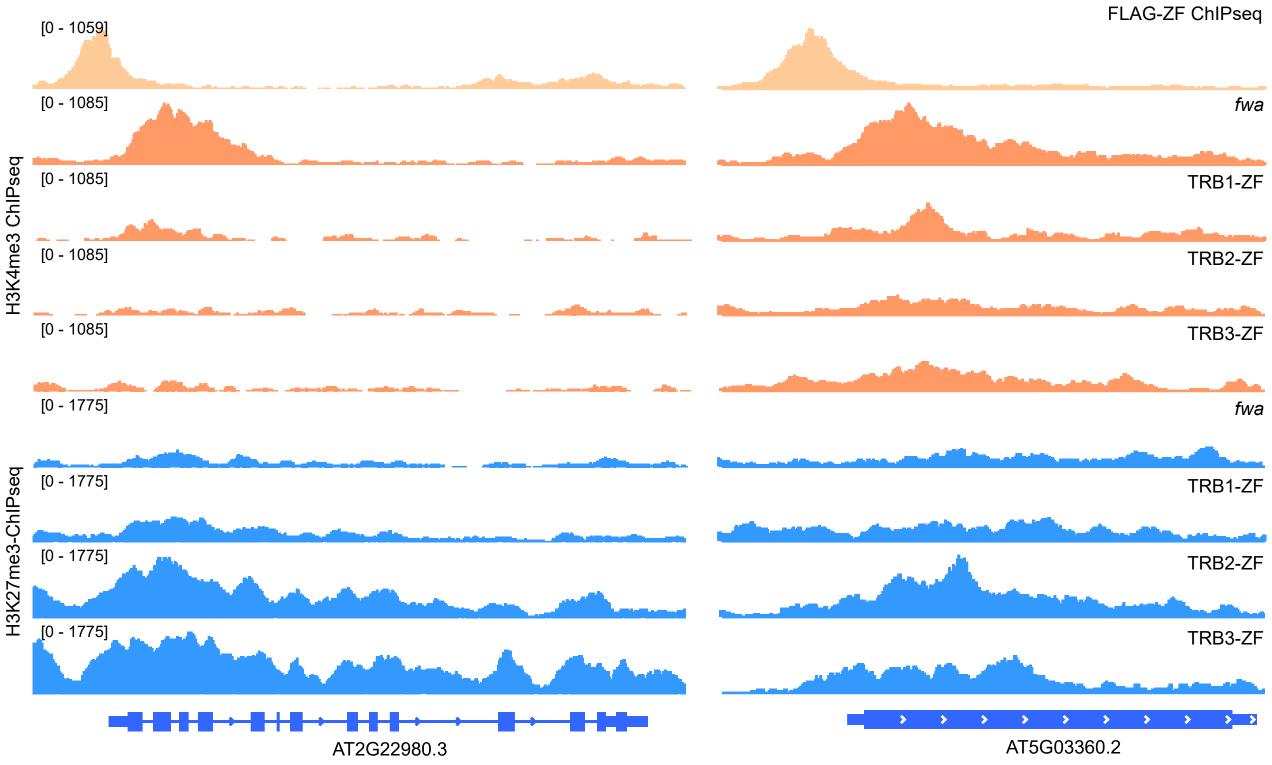

Supplementary Fig. 14 TRB-ZF fusions triggered H3K4me3 demethylation and H3K27me3 deposition over two representative ZF off-target sites. Screenshots of H3K4me3 and H3K27me3 ChIP-seq signals in *fwa*, TRB1-ZF, TRB2-ZF, and TRB3-ZF over two representative ZF off target sites. FLAG-ZF ChIP-seq indicates the ZF binding sites.

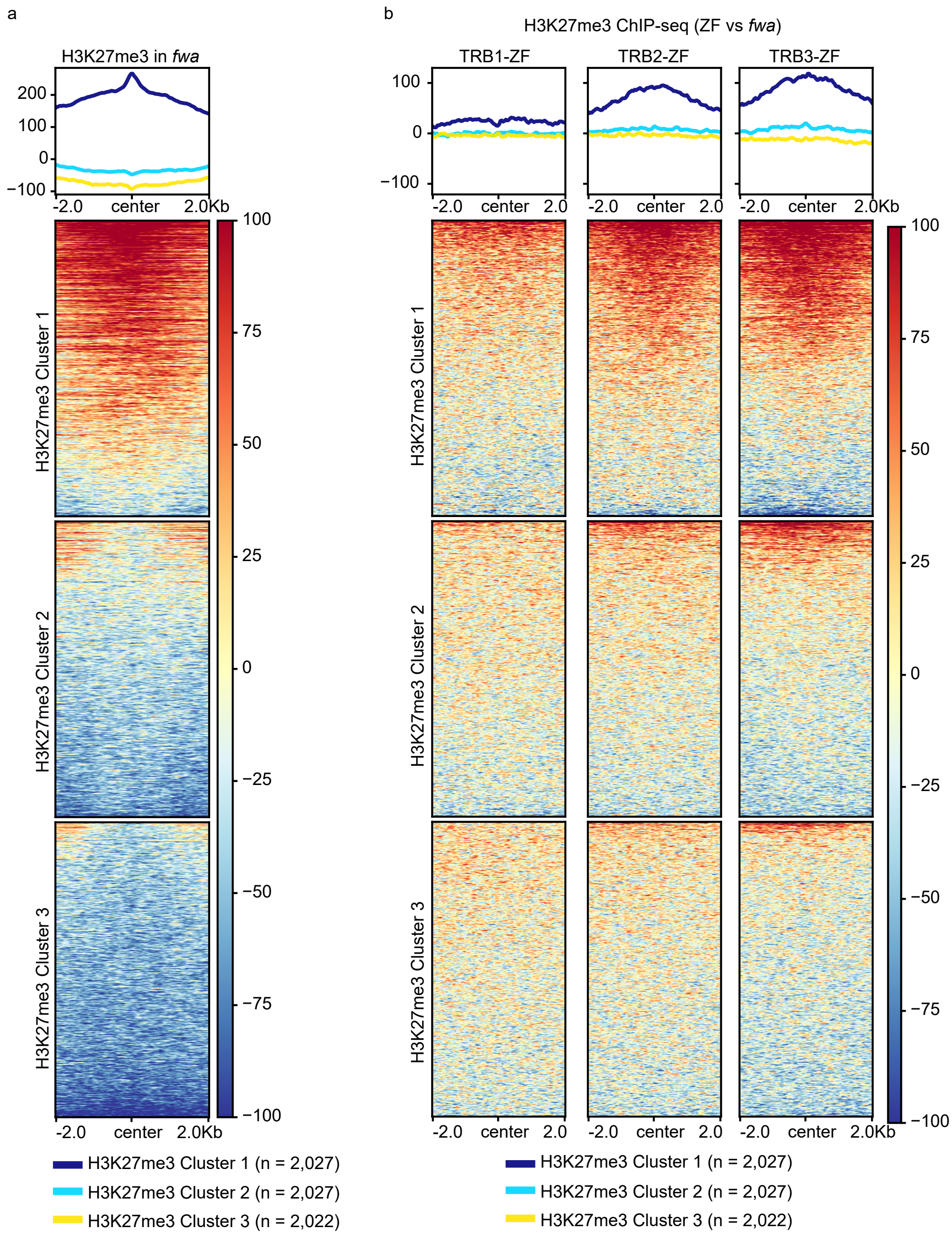

Supplementary Fig. 15 TRB-ZFs triggered H3K27me3 deposition mainly occurs at ZF off-target sites with high levels of pre-existing H3K27me3. a Metaplot and heatmaps representing the normalized H3K27me3 ChIP-seq signals in *fwa* plant over three clusters of ZF off-target sites with high (H3K27me3 Cluster1, n=2,027,), medium (H3K27me3 Cluster2, n=2,027), or low (H3K27me3 Cluster3, n=2,022) levels of pre-existing H3K27me3. **b** Metaplots and heatmaps showing the normalized H3K27me3 ChIP-seq signals in TRB-ZFs versus *fwa* over three H3K27me3 clusters.

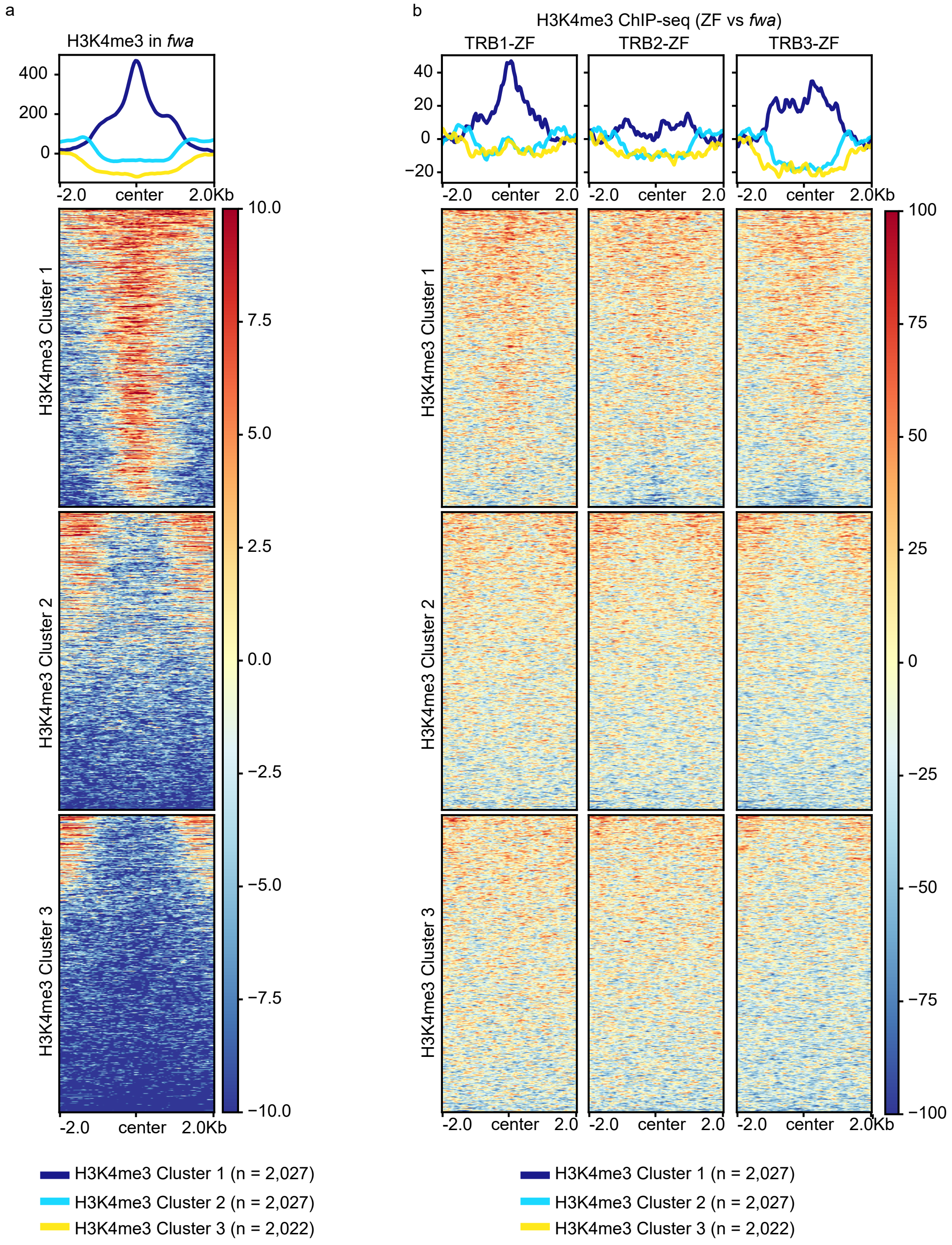

Supplementary Fig. 16 TRB-ZFs triggered H3K4me3 removal mainly occurs at ZF off-target sites with medium or low levels of pre-existing H3K4me3. a Metaplots and heatmaps showing the normalized H3K4me3 ChIP-seq signals in *fwa* over three clusters of ZF off-target sites with high (H3K4me3 Cluster1, n=2,027), medium (H3K4me3 Cluster2, n=2,027), or low (H3K4me3 Cluster3, n=2,022) levels of pre-existing H3K4me3. **b** Metaplots and heatmaps showing the normalized H3K4me3 ChIP-seq signals in TRB-ZFs versus *fwa* over the three H3K4me3 clusters.

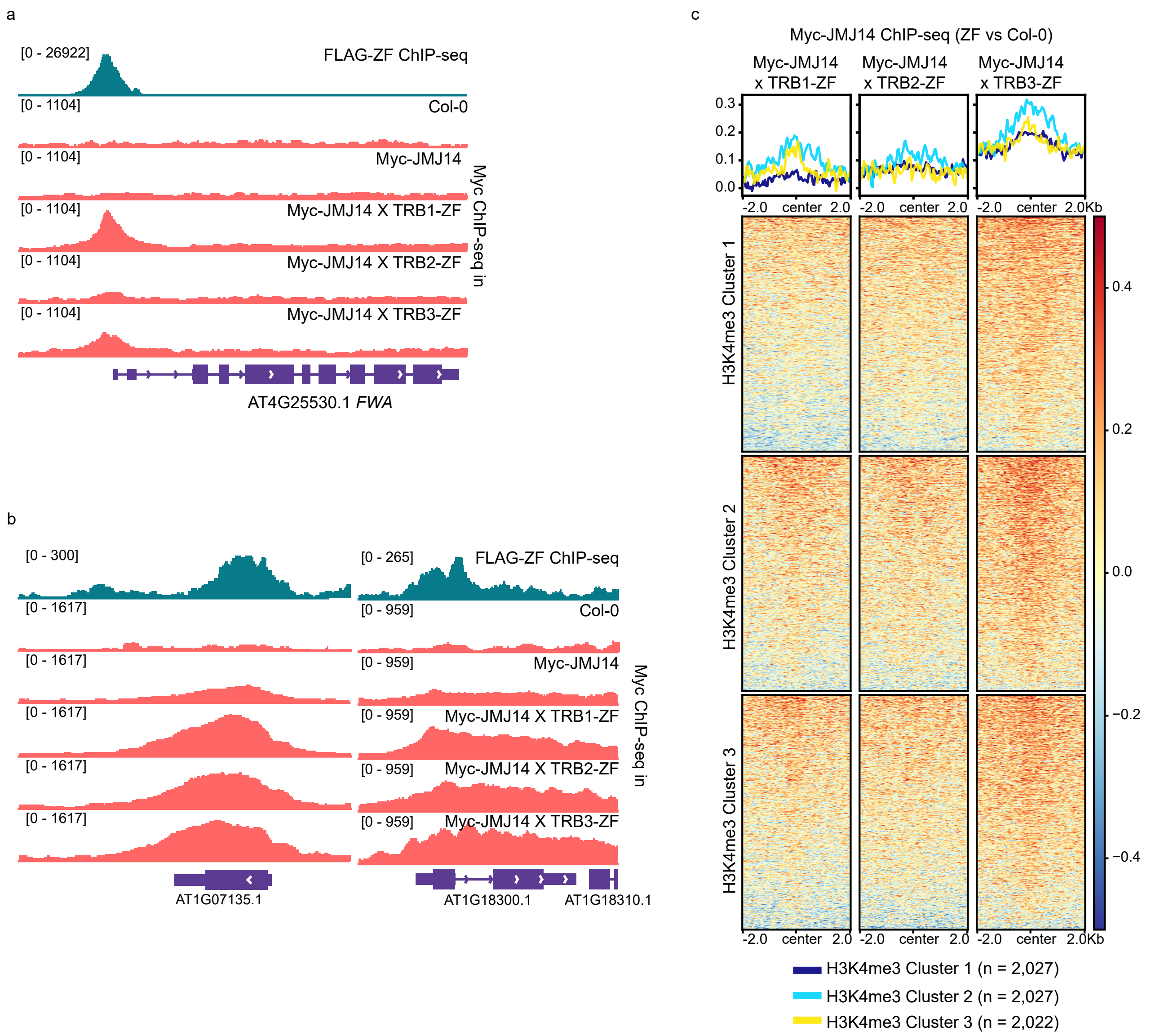

Supplementary Fig. 17 The recruitment of JMJ14 by TRB-ZFs mainly occurs at the ZF off-target sites with medium or low levels of pre-existing H3K4me3. a-b Screenshots of Myc ChIP-seq in Col-0 plant, Myc-JMJ14 transgenic lines in Col-0, TRB1-ZF, TRB2-ZF, and TRB3-ZF backgrounds at *FWA* (**a**) and two representative ZF off-target genes (**b**). FLAG-ZF ChIP-seq indicates the ZF binding site. **c** Normalized Myc-JMJ14 ChIP-seq signals over three H3K4me3 Clusters of ZF off-target sites in Myc-JMJ14 x TRB-ZFs crossed lines versus Myc-JMJ14 transgenic line in Col-0 background.

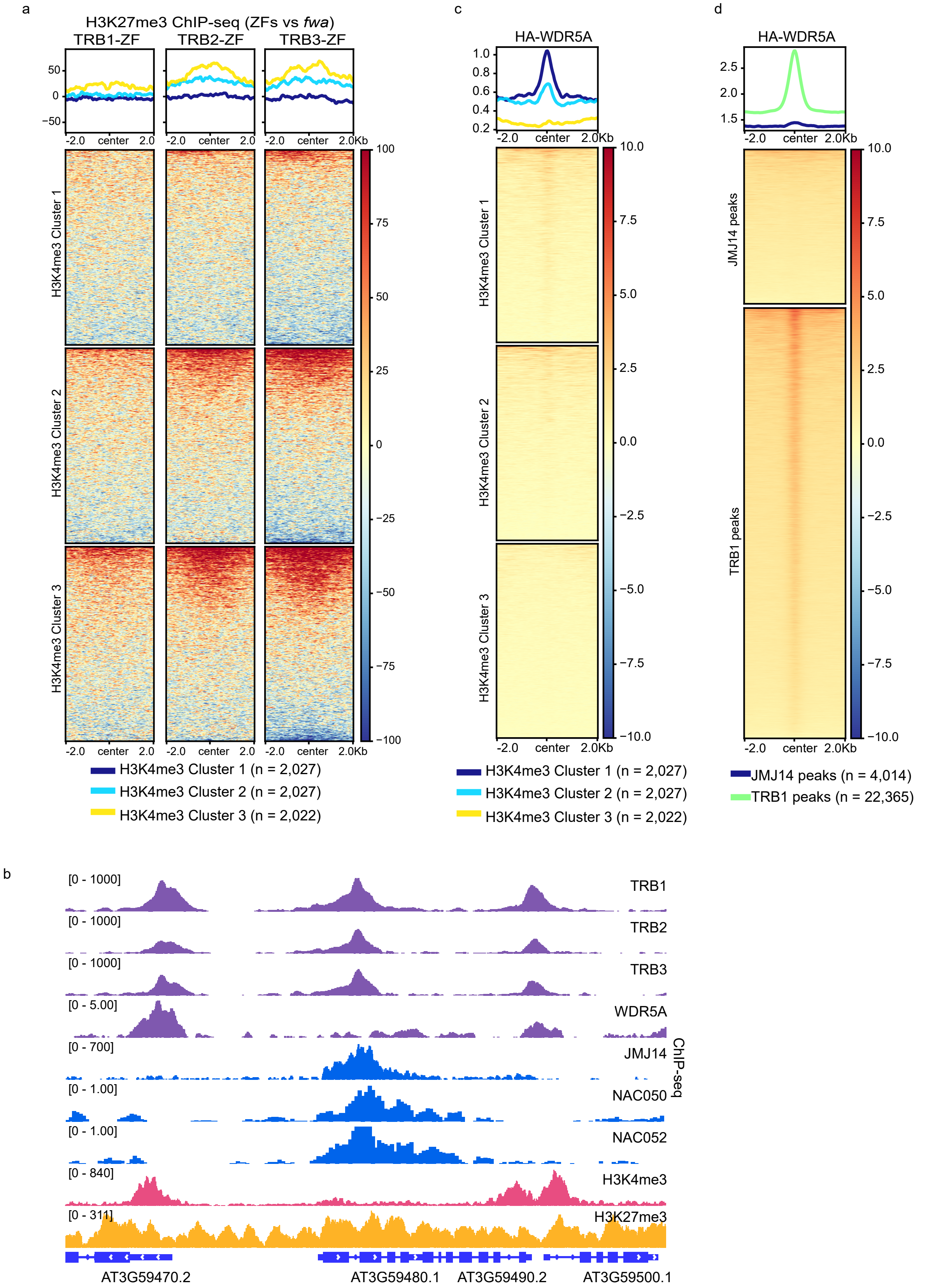

Supplementary Fig. 18 TRB-ZF might recruit WDR5A to add H3K4me3 to ZF off-target sites with high levels of pre-existing H3K4me3. a H3K27me3 ChIP-seq in TRB-ZFs versus *fwa* over three H3K4me3 clusters of ZF off-target sites. **b** Screenshots representing the ChIP-seq of FLAG-TRB1, FLAG-TRB2, FLAG-TRB3, FLAG-JMJ14, HA-WDR5A, HA-NAC050, HA-NAC052, H3K4me3, and H3K27me3 over a representative locus. **c-d** Metaplots and heatmaps depicting HA-WDR5A ChIP-seq signals over three H3K4me3 clusters of ZF off-target sites (**c**), or over JMJ14 peaks (n=4,014) or TRB1 peaks (n=22,36) (**d**).
