# Supplementary material for: Arabidopsis TRB proteins function in H3K4me3 demethylation by recruiting JMJ14
Source: Nat Commun. 2023 Mar 28;14:1736. doi: 10.1038/s41467-023-37263-9 (PMC10049986; doi:10.1038/s41467-023-37263-9)
Supplement: Supplementary file 3 — Description of Additional Supplementary Files [file 41467_2023_37263_MOESM3_ESM.pdf]

Description of Additional Supplementary Files:

Supplementary Dataset 1: FLAG-TRBs IP-MS data.

Supplementary Dataset 2: Diff-H3K4me3 and H3K27me3 regions in trb1/2/3 and jmj14-1 mutants versus Col-0.

Supplementary Dataset 3: The list of TRB1 and JMJ14 co-bound genes.

Supplementary Dataset 4: The H3K4me3 and H3K27me3 levels of TRB1- JMJ14 co-bound genes in trb1/2/3 mutant vs Col-0. 5)

Supplementary Dataset 5: The DEG list of trb1/2/3 and jmj14-1 mutants.

Supplementary Dataset 6: List of primers.
