# Supplementary material for: Arabidopsis TRB proteins function in H3K4me3 demethylation by recruiting JMJ14
Source: Nat Commun. 2023 Mar 28;14:1736. doi: 10.1038/s41467-023-37263-9 (PMC10049986; doi:10.1038/s41467-023-37263-9)
Supplement: Supplementary file 10 — Reporting Summary [file 41467_2023_37263_MOESM10_ESM.pdf]

nature portfolio

Corresponding author(s): Steven E Jacobsen

Last updated by author(s): Ming Wang, Zhenhui Zhong

Reporting Summary

Nature Portfolio wishes to improve the reproducibility of the work that we publish. This form provides structure for consistency and transparency in reporting. For further information on Nature Portfolio policies, see our Editorial Policies and the Editorial Policy Checklist.

Statistics

For all statistical analyses, confirm that the following items are present in the figure legend, table legend, main text, or Methods section.

n/a	Confirmed	
☐	☒	The exact sample size (n) for each experimental group/condition, given as a discrete number and unit of measurement
☒	☐	A statement on whether measurements were taken from distinct samples or whether the same sample was measured repeatedly
☐	☒	The statistical test(s) used AND whether they are one- or two-sided<br>*Only common tests should be described solely by name; describe more complex techniques in the Methods section.*
☒	☐	A description of all covariates tested
☒	☐	A description of any assumptions or corrections, such as tests of normality and adjustment for multiple comparisons
☐	☒	A full description of the statistical parameters including central tendency (e.g. means) or other basic estimates (e.g. regression coefficient) AND variation (e.g. standard deviation) or associated estimates of uncertainty (e.g. confidence intervals)
☐	☒	For null hypothesis testing, the test statistic (e.g. F, t, r) with confidence intervals, effect sizes, degrees of freedom and P value noted<br>*Give P values as exact values whenever suitable.*
☒	☐	For Bayesian analysis, information on the choice of priors and Markov chain Monte Carlo settings
☒	☐	For hierarchical and complex designs, identification of the appropriate level for tests and full reporting of outcomes
☐	☒	Estimates of effect sizes (e.g. Cohen's d, Pearson's r), indicating how they were calculated

Our web collection on statistics for biologists contains articles on many of the points above.

Software and code

Policy information about availability of computer code

Data collection	No software was used for data collection.
Data analysis	RNA-seq analysis<br>Cleaned short reads were aligned to reference genome tair10 by Bowtie2 (v2.1.0), and expression abundance was calculated by RSEM with default parameters. Heatmaps were visualized with the R package pheatmap. To reduce false positive of differential expression, transcripts with less than 5 reads of all replicates in total were regarded as lowly expressed genes and have been removed in subsequent analysis. Differential expression analysis was conducted using edgeR. A threshold of p value < 0.05 and Fold Change > 2 were used to decide whether significant expression difference exists between samples.<br><br>ChIP-seq analysis<br>ChIP-seq fastq reads were aligned to the TAIR10 reference genome with Bowtie (v1.1.2), allowing only uniquely mapping reads with 0 mismatches. Duplicated reads were removed by Samtools. ChIP-seq peaks were called by MACS2 (v2.1.1) and annotated with ChIPseeker. Differential peaks were called by bdgdiff function in MACS2. ChIP-seq data metaplots were plotted by deeptools (v2.5.1). For Pol II 5’ occupancy analysis, Pol II occupancy was calculated based normalized reads count (RPKM) on a TSS +/- 200 bp region and a TSS +500 bp to TTS gene body region by bedtools. Detailed information for published ChIP-seq datasets is listed in Supplementary Table 2.<br><br>Whole genome bisulfite sequencing (BS-seq) analysis<br>Trim_galore (http://www.bioinformatics.babraham.ac.uk/projects/trim_galore/) was used to trim adapters after filtering low quality reads. BS-seq reads were aligned to TAIR10 reference genome by Bismark (v0.18.2) with default settings. Reads with three or more consecutive CHH sites were considered as unconverted reads and filtered. DNA methylation levels were defined as #C/ (#C + #T). DMRs (Differentially Methylated Regions) were called by DMRcaller with p < 0.01 for where the differences in CG, CHG, and CHH methylation were at least 0.4, 0.2, and 0.1, respectively.

BS-PCR analysis
BS-PCR data were trimmed with primer sequences and mapped to TAIR10 reference genome with bsmap (v2.90) allowing 2 mismatches and 1 best hit (-v 2 -w 1).

For manuscripts utilizing custom algorithms or software that are central to the research but not yet described in published literature, software must be made available to editors and reviewers. We strongly encourage code deposition in a community repository (e.g. GitHub). See the Nature Portfolio guidelines for submitting code & software for further information.

Data

Policy information about availability of data

All manuscripts must include a data availability statement. This statement should provide the following information, where applicable:
- Accession codes, unique identifiers, or web links for publicly available datasets
- A description of any restrictions on data availability
- For clinical datasets or third party data, please ensure that the statement adheres to our policy

All high-throughput sequencing data generated in this study are accessible at NCBI's Gene Expression Omnibus (GEO) via GEO Series accession number GSE204681 (https://www.ncbi.nlm.nih.gov/geo/query/acc.cgi?acc=GSE204681). The mass spectrometry proteomics data have been deposited to the ProteomeXchange Consortium via the MassIVE partner repository, and the accession number is MSV000091349 (https://massive.ucsd.edu/ProteoSAFe/dataset_files.jsp?task=5003a58b4c39487d8bd2b0e508388a4c#%7B%22table_sort_history%22%3A%22main.collection_asc%22%7D).

Field-specific reporting

Please select the one below that is the best fit for your research. If you are not sure, read the appropriate sections before making your selection.

☒ Life sciences ☐ Behavioural & social sciences ☐ Ecological, evolutionary & environmental sciences

For a reference copy of the document with all sections, see nature.com/documents/nr-reporting-summary-flat.pdf

Life sciences study design

All studies must disclose on these points even when the disclosure is negative.

Sample size	No sample size calculation was performed.
Data exclusions	No data exclusion in the study.
Replication	Two replicates for ChIP-seq. Three replicates for RNA-seq samples. Two replicates for WGBS data. Two replicates for BS-PCR.
Randomization	For all experiments, treatment and control samples were grown side by side, each replicate on separate plate.
Blinding	No blinding used.

Reporting for specific materials, systems and methods

We require information from authors about some types of materials, experimental systems and methods used in many studies. Here, indicate whether each material, system or method listed is relevant to your study. If you are not sure if a list item applies to your research, read the appropriate section before selecting a response.

Materials & experimental systems

n/a	Involved in the study	
☐	☒	Antibodies
☒	☐	Eukaryotic cell lines
☒	☐	Palaeontology and archaeology
☒	☐	Animals and other organisms
☒	☐	Human research participants
☒	☐	Clinical data
☒	☐	Dual use research of concern

Methods

n/a	Involved in the study	
☐	☒	ChIP-seq
☒	☐	Flow cytometry
☒	☐	MRI-based neuroimaging

Antibodies

Antibodies used	Anti-H3K27me3 (Millipore Sigma)<br>Anti-H3 (Abcam)

	Anti-H3K4me3 (Millipore Sigma)<br>Anti-FLAG M2 (Sigma)<br>Anti-Myc (Cell Signaling)
Validation	anti-FLAG M2 (Sigma): The antibody is validated by https://www.sigmaaldrich.com/catalog/product/sigma/f1804<br>anti-H3 (Ab1791, Abcam): The antibody is validated by https://www.abcam.com/histone-h3-antibody-nuclear-marker-and-chip-grade-ab1791.html<br>Anti-H3K27me3 (Millipore Sigma): The antibody is validated by https://www.emdmillipore.com/US/en/product/Anti-trimethyl-Histone-H3-Lys27-Antibody,MM_NF-07-449<br>Anti-H3K4me3 (Millipore Sigma): The antibody is validated by https://www.emdmillipore.com/US/en/product/Anti-trimethyl-Histone-H3-Lys4-Antibody,MM_NF-07-473<br>Anti-Myc (Cell Signaling): The antibody is validated by https://www.cellsignal.com/products/primary-antibodies/myc-tag-9b11-mouse-mab/2276

ChIP-seq

Data deposition

- [x] Confirm that both raw and final processed data have been deposited in a public database such as GEO.
- [x] Confirm that you have deposited or provided access to graph files (e.g. BED files) for the called peaks.

Data access links<br>*May remain private before publication.*	All high-throughput sequencing data generated in this study are accessible at NCBI's Gene Expression Omnibus (GEO) via GEO Series accession number GSE204681(https://www.ncbi.nlm.nih.gov/geo/query/acc.cgi?acc=GSE204681).
Files in database submission	RNAseq-fwa-Rep1.bw<br>RNAseq-fwa-Rep2.bw<br>RNAseq-fwa-Rep3.bw<br>RNAseq-TRB1-ZF-Rep1.bw<br>RNAseq-TRB1-ZF-Rep2.bw<br>RNAseq-TRB2-ZF-Rep1.bw<br>RNAseq-TRB2-ZF-Rep2.bw<br>RNAseq-TRB2-ZF-Rep3.bw<br>RNAseq-TRB3-ZF-Rep1.bw<br>RNAseq-TRB3-ZF-Rep2.bw<br>RNAseq-TRB3-ZF-Rep3.bw<br>RNAseq-Col0-Rep1.bw<br>RNAseq-Col0-Rep2.bw<br>RNAseq-Col0-Rep3.bw<br>RNAseq-jmj14-Rep1.bw<br>RNAseq-jmj14-Rep2.bw<br>RNAseq-jmj14-Rep3.bw<br>RNAseq-trb123-Rep1.bw<br>RNAseq-trb123-Rep2.bw<br>RNAseq-trb123-Rep3.bw<br>H3-ChIPseq-Col0-Rep2_S17_L002.bw<br>H3-ChIPseq-trb123-Rep2_S16_L002.bw<br>H3K4me3-ChIPseq-Col0-Rep2_S20_L002.bw<br>H3K4me3-ChIPseq-trb123-Rep2_S19_L002.bw<br>H3-ChIPseq-jmj14-Rep1.bw<br>H3K4me3-ChIPseq-Col0-Rep1.bw<br>H3K4me3-ChIPseq-jmj14-Rep1.bw<br>H3K4me3-ChIPseq-trb123-Rep1.bw<br>FLAG-ChIPseq-Col0-Rep1.bw<br>FLAG-ChIPseq-Col0-Rep2.bw<br>FLAG-ChIPseq-JMJ14-Rep1.bw<br>FLAG-ChIPseq-JMJ14-Rep2.bw<br>H3-ChIPseq-Col0-Rep1.bw<br>H3-ChIPseq-Col0-Rep2.bw<br>H3-ChIPseq-trb123-Rep1.bw<br>H3-ChIPseq-trb123-Rep2.bw<br>H3K27me3-ChIPseq-Col0-Rep1.bw<br>H3K27me3-ChIPseq-Col0-Rep2.bw<br>H3K27me3-ChIPseq-trb123-Rep1.bw<br>H3K27me3-ChIPseq-trb123-Rep2.bw<br>FLAG-ChIPseq-TRB1-Rep1.bw<br>FLAG-ChIPseq-TRB1-Rep2.bw<br>FLAG-ChIPseq-TRB2-Rep1.bw<br>FLAG-ChIPseq-TRB2-Rep2.bw

FLAG-ChIPseq-TRB3-Rep1.bw
FLAG-ChIPseq-TRB3-Rep2.bw
H3-ChIPseq-fwa-Rep2.bw
H3-ChIPseq-TRB1-ZF-Rep2.bw
H3-ChIPseq-TRB2-ZF-Rep2.bw
H3-ChIPseq-TRB3-ZF-Rep2.bw
H3K27me3-ChIPseq-fwa-Rep2.bw
H3K27me3-ChIPseq-TRB1-ZF-Rep2.bw
H3K27me3-ChIPseq-TRB2-ZF-Rep2.bw
H3K27me3-ChIPseq-TRB3-ZF-Rep2.bw
H3K4me3-ChIPseq-fwa-Rep2.bw
H3K4me3-ChIPseq-TRB1-ZF-Rep2.bw
H3K4me3-ChIPseq-TRB2-ZF-Rep2.bw
H3K4me3-ChIPseq-TRB3-ZF-Rep2.bw
H3-ChIPseq-fwa-Rep1.bw
H3-ChIPseq-fwa-Rep3.bw
H3-ChIPseq-TRB1-ZF-Rep1.bw
H3-ChIPseq-TRB1-ZF-Rep3.bw
H3_TRB2_ZF.bw
H3_TRB3_ZF.bw
H3K27me3-ChIPseq-fwa-Rep1.bw
H3K27me3-ChIPseq-TRB1-ZF-Rep1.bw
H3K27me3-ChIPseq-TRB2-ZF-Rep1.bw
H3K27me3-ChIPseq-TRB3-ZF-Rep1.bw
H3K4me3-ChIPseq-fwa-Rep1.bw
H3K4me3-ChIPseq-fwa-Rep3.bw
H3K4me3-ChIPseq-TRB1-ZF-Rep1.bw
H3K4me3-ChIPseq-TRB1-ZF-Rep3.bw
H3K4me3-ChIPseq-TRB2-ZF-Rep1.bw
H3K4me3-ChIPseq-TRB3-ZF-Rep1.bw
FLAG-Col-0-Rep3.bw
FLAG-Col-0-Rep4.bw
FLAG-TRB1-rep3.bw
FLAG-TRB1-rep4.bw
FLAG-TRB2-rep3.bw
FLAG-TRB2-rep4.bw
FLAG-TRB3-rep3.bw
FLAG-TRB3-rep4.bw
MYC-Col-0_S13_L003.bw
MYC-JMJ14-ZF-TRB1_S15_L003.bw
MYC-JMJ14-ZF-TRB1_S18_L003.bw
MYC-JMJ14-ZF-TRB2_S16_L003.bw
MYC-JMJ14-ZF-TRB3_S17_L003.bw
MYC-JMJ14_S14_L003.bw
input-Col-0_S23_L003.bw
input-JMJ14-ZF-TRB1_S25_L003.bw
input-JMJ14-ZF-TRB1_S28_L003.bw
input-JMJ14-ZF-TRB2_S26_L003.bw
input-JMJ14-ZF-TRB3_S27_L003.bw
input-JMJ14_S24_L003.bw
FLAG-TRB1-rep3_peaks.narrowPeak
FLAG-TRB1-rep4_peaks.narrowPeak
FLAG-TRB2-rep3_peaks.narrowPeak
FLAG-TRB2-rep4_peaks.narrowPeak
FLAG-TRB3-rep3_peaks.narrowPeak
FLAG-TRB3-rep4_peaks.narrowPeak
MYC-JMJ14-ZF-TRB1_S15_L003_peaks.narrowPeak
MYC-JMJ14-ZF-TRB1_S18_L003_peaks.narrowPeak
MYC-JMJ14-ZF-TRB2_S16_L003_peaks.narrowPeak
MYC-JMJ14-ZF-TRB3_S17_L003_peaks.narrowPeak
MYC-JMJ14_S14_L003_peaks.narrowPeak
H3K4me3-ChIPseq-Col0-Rep2_peaks.narrowPeak
H3K4me3-ChIPseq-trb123-Rep2_peaks.narrowPeak
H3K4me3-ChIPseq-Col0-Rep1_peaks.narrowPeak
H3K4me3-ChIPseq-jmj14-Rep1_peaks.narrowPeak
H3K4me3-ChIPseq-trb123-Rep1_peaks.narrowPeak
FLAG-ChIPseq-JMJ14-Rep1_peaks.narrowPeak
FLAG-ChIPseq-JMJ14-Rep2_peaks.narrowPeak

H3K27me3-ChIPseq-Col0-Rep1_peaks.narrowPeak
H3K27me3-ChIPseq-Col0-Rep2_peaks.narrowPeak
H3K27me3-ChIPseq-trb123-Rep1_peaks.narrowPeak
H3K27me3-ChIPseq-trb123-Rep2_peaks.narrowPeak
FLAG-ChIPseq-TRB1-Rep1_peaks.narrowPeak
FLAG-ChIPseq-TRB1-Rep2_peaks.narrowPeak
FLAG-ChIPseq-TRB2-Rep1_peaks.narrowPeak
FLAG-ChIPseq-TRB2-Rep2_peaks.narrowPeak
FLAG-ChIPseq-TRB3-Rep1_peaks.narrowPeak
FLAG-ChIPseq-TRB3-Rep2_peaks.narrowPeak
H3K27me3-ChIPseq-fwa-Rep2_peaks.narrowPeak
H3K27me3-ChIPseq-TRB1-ZF-Rep2_peaks.narrowPeak
H3K27me3-ChIPseq-TRB2-ZF-Rep2_peaks.narrowPeak
H3K27me3-ChIPseq-TRB3-ZF-Rep2_peaks.narrowPeak
H3K4me3-ChIPseq-fwa-Rep2_peaks.narrowPeak
H3K4me3-ChIPseq-TRB1-ZF-Rep2_peaks.narrowPeak
H3K4me3-ChIPseq-TRB2-ZF-Rep2_peaks.narrowPeak
H3K4me3-ChIPseq-TRB3-ZF-Rep2_peaks.narrowPeak
H3K27me3-ChIPseq-fwa-Rep1_peaks.narrowPeak
H3K27me3-ChIPseq-TRB1-ZF-Rep1_peaks.narrowPeak
H3K27me3-ChIPseq-TRB2-ZF-Rep1_peaks.narrowPeak
H3K27me3-ChIPseq-TRB3-ZF-Rep1_peaks.narrowPeak
H3K4me3-ChIPseq-fwa-Rep1_peaks.narrowPeak
H3K4me3-ChIPseq-fwa-Rep3_peaks.narrowPeak
H3K4me3-ChIPseq-TRB1-ZF-Rep1_peaks.narrowPeak
H3K4me3-ChIPseq-TRB1-ZF-Rep3_peaks.narrowPeak
H3K4me3-ChIPseq-TRB2-ZF-Rep1_peaks.narrowPeak
H3K4me3-ChIPseq-TRB3-ZF-Rep1_peaks.narrowPeak
WGBS-Col0-Rep1_CG.wig
WGBS-Col0-Rep2_CG.wig
WGBS-trb123-Rep1_CG.wig
WGBS-trb123-Rep2_CG.wig
WGBS-Col0-Rep1_CHG.wig
WGBS-Col0-Rep2_CHG.wig
WGBS-trb123-Rep1_CHG.wig
WGBS-trb123-Rep2_CHG.wig
WGBS-Col0-Rep1_CHH.wig
WGBS-Col0-Rep2_CHH.wig
WGBS-trb123-Rep1_CHH.wig
WGBS-trb123-Rep2_CHH.wig
BS-PCR-seq-TRB1-ZF-Rep1_steve_methratio.txt
BS-PCR-seq-TRB2-ZF-Rep1_steve_methratio.txt
BS-PCR-seq-TRB3-ZF-Rep1_steve_methratio.txt
BS-PCRseq-Col0-Rep1_steve_methratio.txt
BS-PCRseq-Col0-Rep2_steve_methratio.txt
BS-PCRseq-fwa-Rep1_steve_methratio.txt
BS-PCRseq-fwa-Rep2_steve_methratio.txt
BS-PCRseq-TRB1-ZF-Rep2_steve_methratio.txt
BS-PCRseq-TRB2-ZF-Rep2_steve_methratio.txt
BS-PCRseq-TRB3-ZF-Rep2_steve_methratio.txt
BS-PCR-seq-TRB1-ZF-Rep1_steve_methratio_type.txt
BS-PCR-seq-TRB2-ZF-Rep1_steve_methratio_type.txt
BS-PCR-seq-TRB3-ZF-Rep1_steve_methratio_type.txt
BS-PCRseq-Col0-Rep1_steve_methratio_type.txt
BS-PCRseq-Col0-Rep2_steve_methratio_type.txt
BS-PCRseq-fwa-Rep1_steve_methratio_type.txt
BS-PCRseq-fwa-Rep2_steve_methratio_type.txt
BS-PCRseq-TRB1-ZF-Rep2_steve_methratio_type.txt
BS-PCRseq-TRB2-ZF-Rep2_steve_methratio_type.txt
BS-PCRseq-TRB3-ZF-Rep2_steve_methratio_type.txt
RNAseq-fwa-Rep1.fastq.gz
RNAseq-fwa-Rep2.fastq.gz
RNAseq-fwa-Rep3.fastq.gz
RNAseq-TRB1-ZF-Rep1.fastq.gz
RNAseq-TRB1-ZF-Rep2.fastq.gz
RNAseq-TRB2-ZF-Rep1.fastq.gz
RNAseq-TRB2-ZF-Rep2.fastq.gz
RNAseq-TRB2-ZF-Rep3.fastq.gz

RNAseq-TRB3-ZF-Rep1.fastq.gz
RNAseq-TRB3-ZF-Rep2.fastq.gz
RNAseq-TRB3-ZF-Rep3.fastq.gz
FLAG-ChIPseq-TRB1-Rep1.fastq.gz
FLAG-ChIPseq-TRB1-Rep2.fastq.gz
FLAG-ChIPseq-TRB2-Rep1.fastq.gz
FLAG-ChIPseq-TRB2-Rep2.fastq.gz
FLAG-ChIPseq-TRB3-Rep1.fastq.gz
FLAG-ChIPseq-TRB3-Rep2.fastq.gz
WGBS-Col0-Rep1_S9_L001_R1_001.fastq.gz
WGBS-Col0-Rep2_S4_L001_R1_001.fastq.gz
WGBS-trb123-Rep1_S5_L001_R1_001.fastq.gz
WGBS-trb123-Rep2_S10_L001_R1_001.fastq.gz
BS-PCRseq-Col0-Rep2.fastq.gz
BS-PCRseq-fwa-Rep2.fastq.gz
BS-PCRseq-TRB1-ZF-Rep2.fastq.gz
BS-PCRseq-TRB2-ZF-Rep2.fastq.gz
BS-PCRseq-TRB3-ZF-Rep2.fastq.gz
BS-PCR-seq-TRB1-ZF-Rep1_S16_L001_R1_001.fastq.gz
BS-PCR-seq-TRB2-ZF-Rep1_S17_L001_R1_001.fastq.gz
BS-PCR-seq-TRB3-ZF-Rep1_S18_L001_R1_001.fastq.gz
BS-PCRseq-Col0-Rep1_S14_L001_R1_001.fastq.gz
BS-PCRseq-fwa-Rep1_S13_L001_R1_001.fastq.gz
RNAseq-Col0-Rep1_S1_L002_R1_001.fastq.gz
RNAseq-Col0-Rep2_S4_L002_R1_001.fastq.gz
RNAseq-Col0-Rep3_S2_L002_R1_001.fastq.gz
RNAseq-jmj14-Rep1_S12_L002_R1_001.fastq.gz
RNAseq-jmj14-Rep2_S5_L002_R1_001.fastq.gz
RNAseq-jmj14-Rep3_S3_L002_R1_001.fastq.gz
RNAseq-trb123-Rep1_S11_L002_R1_001.fastq.gz
RNAseq-trb123-Rep2_S17_L002_R1_001.fastq.gz
RNAseq-trb123-Rep3_S18_L002_R1_001.fastq.gz
H3-ChIPseq-Col0-Rep2_S17_L002_R1_001.fastq.gz
H3-ChIPseq-trb123-Rep2_S16_L002_R1_001.fastq.gz
H3K4me3-ChIPseq-Col0-Rep2_S20_L002_R1_001.fastq.gz
H3K4me3-ChIPseq-trb123-Rep2_S19_L002_R1_001.fastq.gz
H3-ChIPseq-jmj14_S11_L002_R1_001.fastq.gz
H3K4me3-ChIPseq-Col0_S18_L002_R1_001.fastq.gz
H3K4me3-ChIPseq-jmj14_S20_L002_R1_001.fastq.gz
H3K4me3-ChIPseq-trb123_S19_L002_R1_001.fastq.gz
FLAG-ChIPseq-Col0-Rep1_S4_L001_R1_001.fastq.gz
FLAG-ChIPseq-Col0-Rep2_S2_L001_R1_001.fastq.gz
FLAG-ChIPseq-JMJ14-Rep1_S6_L001_R1_001.fastq.gz
FLAG-ChIPseq-JMJ14-Rep2_S7_L001_R1_001.fastq.gz
H3-ChIPseq-Col0-Rep1_S17_L004_R1_001.fastq.gz
H3-ChIPseq-Col0-Rep2_S15_L004_R1_001.fastq.gz
H3-ChIPseq-trb123-Rep1_S20_L004_R1_001.fastq.gz
H3-ChIPseq-trb123-Rep2_S19_L004_R1_001.fastq.gz
H3K27me3-ChIPseq-Col0-Rep1_S38_L004_R1_001.fastq.gz
H3K27me3-ChIPseq-Col0-Rep2_S34_L004_R1_001.fastq.gz
H3K27me3-ChIPseq-trb123-Rep1_S37_L004_R1_001.fastq.gz
H3K27me3-ChIPseq-trb123-Rep2_S35_L004_R1_001.fastq.gz
H3-ChIPseq-fwa-Rep2_L003_R2_001.fastq.gz
H3-ChIPseq-TRB1-ZF-Rep2_S30_L003_R1_001.fastq.gz
H3-ChIPseq-TRB2-ZF-Rep2_S18_L003_R1_001.fastq.gz
H3-ChIPseq-TRB3-ZF-Rep2_S21_L003_R1_001.fastq.gz
H3K27me3-ChIPseq-fwa-Rep2_S50_L003_R1_001.fastq.gz
H3K27me3-ChIPseq-TRB1-ZF-Rep2_S53_L003_R1_001.fastq.gz
H3K27me3-ChIPseq-TRB2-ZF-Rep2_S75_L003_R1_001.fastq.gz
H3K27me3-ChIPseq-TRB3-ZF-Rep2_S89_L003_R1_001.fastq.gz
H3K4me3-ChIPseq-fwa-Rep2_S45_L003_R1_001.fastq.gz
H3K4me3-ChIPseq-TRB1-ZF-Rep2_S84_L003_R1_001.fastq.gz
H3K4me3-ChIPseq-TRB2-ZF-Rep2_S82_L003_R1_001.fastq.gz
H3K4me3-ChIPseq-TRB3-ZF-Rep2_S81_L003_R1_001.fastq.gz
H3-ChIPseq-fwa-Rep1_S8_L001_R1_001.fastq.gz
H3-ChIPseq-fwa-Rep3_S13_L004_R1_001.fastq.gz
H3-ChIPseq-TRB1-ZF-Rep1_S5_L001_R1_001.fastq.gz
H3-ChIPseq-TRB1-ZF-Rep3_S14_L004_R1_001.fastq.gz

H3-ChIPseq-TRB2-ZF-Rep1_S4_L001_R1_001.fastq.gz
H3-ChIPseq-TRB3-ZF-Rep1_S1_L001_R1_001.fastq.gz
H3K27me3-ChIPseq-fwa-Rep1_S18_L001_R1_001.fastq.gz
H3K27me3-ChIPseq-TRB1-ZF-Rep1_S23_L001_R1_001.fastq.gz
H3K27me3-ChIPseq-TRB2-ZF-Rep1_S21_L001_R1_001.fastq.gz
H3K27me3-ChIPseq-TRB3-ZF-Rep1_S22_L001_R1_001.fastq.gz
H3K4me3-ChIPseq-fwa-Rep1_S11_L001_R1_001.fastq.gz
H3K4me3-ChIPseq-fwa-Rep3_S15_L004_R1_001.fastq.gz
H3K4me3-ChIPseq-TRB1-ZF-Rep1_S17_L001_R1_001.fastq.gz
H3K4me3-ChIPseq-TRB1-ZF-Rep3_S16_L004_R1_001.fastq.gz
H3K4me3-ChIPseq-TRB2-ZF-Rep1_S13_L001_R1_001.fastq.gz
H3K4me3-ChIPseq-TRB3-ZF-Rep1_S14_L001_R1_001.fastq.gz
BS-PCR-seq-TRB1-ZF-Rep1_S16_L001_R2_001.fastq.gz
BS-PCR-seq-TRB2-ZF-Rep1_S17_L001_R2_001.fastq.gz
BS-PCR-seq-TRB3-ZF-Rep1_S18_L001_R2_001.fastq.gz
BS-PCRseq-Col0-Rep1_S14_L001_R2_001.fastq.gz
BS-PCRseq-fwa-Rep1_S13_L001_R2_001.fastq.gz
RNAseq-Col0-Rep1_S1_L002_R2_001.fastq.gz
RNAseq-Col0-Rep2_S4_L002_R2_001.fastq.gz
RNAseq-Col0-Rep3_S2_L002_R2_001.fastq.gz
RNAseq-jmj14-Rep1_S12_L002_R2_001.fastq.gz
RNAseq-jmj14-Rep2_S5_L002_R2_001.fastq.gz
RNAseq-jmj14-Rep3_S3_L002_R2_001.fastq.gz
RNAseq-trb123-Rep1_S11_L002_R2_001.fastq.gz
RNAseq-trb123-Rep2_S17_L002_R2_001.fastq.gz
RNAseq-trb123-Rep3_S18_L002_R2_001.fastq.gz
H3-ChIPseq-Col0-Rep2_S17_L002_R2_001.fastq.gz
H3-ChIPseq-trb123-Rep2_S16_L002_R2_001.fastq.gz
H3K4me3-ChIPseq-Col0-Rep2_S20_L002_R2_001.fastq.gz
H3K4me3-ChIPseq-trb123-Rep2_S19_L002_R2_001.fastq.gz
H3-ChIPseq-jmj14_S11_L002_R2_001.fastq.gz
H3K4me3-ChIPseq-Col0_S18_L002_R2_001.fastq.gz
H3K4me3-ChIPseq-jmj14_S20_L002_R2_001.fastq.gz
H3K4me3-ChIPseq-trb123_S19_L002_R2_001.fastq.gz
FLAG-ChIPseq-Col0-Rep1_S4_L001_R2_001.fastq.gz
FLAG-ChIPseq-Col0-Rep2_S2_L001_R2_001.fastq.gz
FLAG-ChIPseq-JMJ14-Rep1_S6_L001_R2_001.fastq.gz
FLAG-ChIPseq-JMJ14-Rep2_S7_L001_R2_001.fastq.gz
H3-ChIPseq-Col0-Rep1_S17_L004_R2_001.fastq.gz
H3-ChIPseq-Col0-Rep2_S15_L004_R2_001.fastq.gz
H3-ChIPseq-trb123-Rep1_S20_L004_R2_001.fastq.gz
H3-ChIPseq-trb123-Rep2_S19_L004_R2_001.fastq.gz
H3K27me3-ChIPseq-Col0-Rep1_S38_L004_R2_001.fastq.gz
H3K27me3-ChIPseq-Col0-Rep2_S34_L004_R2_001.fastq.gz
H3K27me3-ChIPseq-trb123-Rep1_S37_L004_R2_001.fastq.gz
H3K27me3-ChIPseq-trb123-Rep2_S35_L004_R2_001.fastq.gz
H3-ChIPseq-fwa-Rep2_S32_L003_R1_001.fastq.gz
H3-ChIPseq-TRB1-ZF-Rep2_S30_L003_R2_001.fastq.gz
H3-ChIPseq-TRB2-ZF-Rep2_S18_L003_R2_001.fastq.gz
H3-ChIPseq-TRB3-ZF-Rep2_S21_L003_R2_001.fastq.gz
H3K27me3-ChIPseq-fwa-Rep2_S50_L003_R2_001.fastq.gz
H3K27me3-ChIPseq-TRB1-ZF-Rep2_S53_L003_R2_001.fastq.gz
H3K27me3-ChIPseq-TRB2-ZF-Rep2_S75_L003_R2_001.fastq.gz
H3K27me3-ChIPseq-TRB3-ZF-Rep2_S89_L003_R2_001.fastq.gz
H3K4me3-ChIPseq-fwa-Rep2_S45_L003_R2_001.fastq.gz
H3K4me3-ChIPseq-TRB1-ZF-Rep2_S84_L003_R2_001.fastq.gz
H3K4me3-ChIPseq-TRB2-ZF-Rep2_S82_L003_R2_001.fastq.gz
H3K4me3-ChIPseq-TRB3-ZF-Rep2_S81_L003_R2_001.fastq.gz
H3-ChIPseq-fwa-Rep1_S8_L001_R2_001.fastq.gz
H3-ChIPseq-fwa-Rep3_S13_L004_R2_001.fastq.gz
H3-ChIPseq-TRB1-ZF-Rep1_S5_L001_R2_001.fastq.gz
H3-ChIPseq-TRB1-ZF-Rep3_S14_L004_R2_001.fastq.gz
H3-ChIPseq-TRB2-ZF-Rep1_S4_L001_R2_001.fastq.gz
H3-ChIPseq-TRB3-ZF-Rep1_S1_L001_R2_001.fastq.gz
H3K27me3-ChIPseq-fwa-Rep1_S18_L001_R2_001.fastq.gz
H3K27me3-ChIPseq-TRB1-ZF-Rep1_S23_L001_R2_001.fastq.gz
H3K27me3-ChIPseq-TRB2-ZF-Rep1_S21_L001_R2_001.fastq.gz
H3K27me3-ChIPseq-TRB3-ZF-Rep1_S22_L001_R2_001.fastq.gz

H3K4me3-ChIPseq-fwa-Rep1_S11_L001_R2_001.fastq.gz
H3K4me3-ChIPseq-fwa-Rep3_S15_L004_R2_001.fastq.gz
H3K4me3-ChIPseq-TRB1-ZF-Rep1_S17_L001_R2_001.fastq.gz
H3K4me3-ChIPseq-TRB1-ZF-Rep3_S16_L004_R2_001.fastq.gz
H3K4me3-ChIPseq-TRB2-ZF-Rep1_S13_L001_R2_001.fastq.gz
H3K4me3-ChIPseq-TRB3-ZF-Rep1_S14_L001_R2_001.fastq.gz
FLAG-Col-0-Rep3_R1_001.fastq.gz
FLAG-Col-0-Rep3_R2_001.fastq.gz
FLAG-Col-0-Rep4_R1_001.fastq.gz
FLAG-Col-0-Rep4_R2_001.fastq.gz
FLAG-TRB1-rep3_R1_001.fastq.gz
FLAG-TRB1-rep3_R2_001.fastq.gz
FLAG-TRB1-rep4_R1_001.fastq.gz
FLAG-TRB1-rep4_R2_001.fastq.gz
FLAG-TRB2-rep3_R1_001.fastq.gz
FLAG-TRB2-rep3_R2_001.fastq.gz
FLAG-TRB2-rep4_R1_001.fastq.gz
FLAG-TRB2-rep4_R2_001.fastq.gz
FLAG-TRB3-rep3_R1_001.fastq.gz
FLAG-TRB3-rep3_R2_001.fastq.gz
FLAG-TRB3-rep4_R1_001.fastq.gz
FLAG-TRB3-rep4_R2_001.fastq.gz
MYC-Col-0_S13_L003_R1_001.fastq.gz
MYC-Col-0_S13_L003_R2_001.fastq.gz
MYC-JMJ14-ZF-TRB1_S15_L003_R1_001.fastq.gz
MYC-JMJ14-ZF-TRB1_S15_L003_R2_001.fastq.gz
MYC-JMJ14-ZF-TRB1_S18_L003_R1_001.fastq.gz
MYC-JMJ14-ZF-TRB1_S18_L003_R2_001.fastq.gz
MYC-JMJ14-ZF-TRB2_S16_L003_R1_001.fastq.gz
MYC-JMJ14-ZF-TRB2_S16_L003_R2_001.fastq.gz
MYC-JMJ14-ZF-TRB3_S17_L003_R1_001.fastq.gz
MYC-JMJ14-ZF-TRB3_S17_L003_R2_001.fastq.gz
MYC-JMJ14_S14_L003_R1_001.fastq.gz
MYC-JMJ14_S14_L003_R2_001.fastq.gz
input-Col-0_S23_L003_R1_001.fastq.gz
input-Col-0_S23_L003_R2_001.fastq.gz
input-JMJ14-ZF-TRB1_S25_L003_R1_001.fastq.gz
input-JMJ14-ZF-TRB1_S25_L003_R2_001.fastq.gz
input-JMJ14-ZF-TRB1_S28_L003_R1_001.fastq.gz
input-JMJ14-ZF-TRB1_S28_L003_R2_001.fastq.gz
input-JMJ14-ZF-TRB2_S26_L003_R1_001.fastq.gz
input-JMJ14-ZF-TRB2_S26_L003_R2_001.fastq.gz
input-JMJ14-ZF-TRB3_S27_L003_R1_001.fastq.gz
input-JMJ14-ZF-TRB3_S27_L003_R2_001.fastq.gz
input-JMJ14_S24_L003_R1_001.fastq.gz
input-JMJ14_S24_L003_R2_001.fastq.gz

Genome browser session
(e.g. UCSC)

Available at GEO

Methodology

Replicates

Two replicates for ChIP-seq. Three replicates for RNA-seq samples. Two replicates for WGBS data. Two replicates for BS-PCR.

Sequencing depth

Name Total_reads Unique_reads Reads_length Reads_type
FLAG-ChIPseq-Col0-Rep1_S4_L001 55575677 45953411 50 PE
FLAG-ChIPseq-Col0-Rep2_S2_L001 55410005 47067741 50 PE
FLAG-ChIPseq-JMJ14-Rep1_S6_L001 53163491 46194604 50 PE
FLAG-ChIPseq-JMJ14-Rep2_S7_L001 51456477 43688143 50 PE
H3-ChIPseq-Col0-Rep1_S17_L004 56310307 41614613 50 PE
H3-ChIPseq-Col0-Rep2_S15_L004 60183339 44979043 50 PE
H3-ChIPseq-Col0-Rep2_S17_L002 17169970 15553287 50 PE
H3-ChIPseq-Col0_S9_L002 37848924 35107648 50 PE
H3-ChIPseq-fwa-Rep1_S32_L003 31559024 27057347 50 PE
H3-ChIPseq-fwa-Rep1_S8_L001 22025700 20443743 50 PE
H3-ChIPseq-fwa-Rep2_S22_L003 34615984 29849162 50 PE
H3-ChIPseq-jmj14_S11_L002 31019042 27343550 50 PE
H3-ChIPseq-TRB1-ZF-Rep1_S29_L003 32068889 27497183 50 PE

H3-ChIPseq-TRB1-ZF-Rep1_S5_L001 23164852 21784542 50 PE
H3-ChIPseq-TRB1-ZF-Rep2_S30_L003 32058861 26393272 50 PE
H3-ChIPseq-trb123-Rep1_S20_L004 52681115 39041971 50 PE
H3-ChIPseq-trb123-Rep2_S16_L002 17534844 15583606 50 PE
H3-ChIPseq-trb123-Rep2_S19_L004 54288481 39544914 50 PE
H3-ChIPseq-trb123_S10_L002 34173266 31179943 50 PE
H3-ChIPseq-TRB2-ZF-Rep1_S31_L003 31812241 27598799 50 PE
H3-ChIPseq-TRB2-ZF-Rep1_S4_L001 24143133 22618113 50 PE
H3-ChIPseq-TRB2-ZF-Rep2_S18_L003 35155705 30874154 50 PE
H3-ChIPseq-TRB3-ZF-Rep1_S15_L003 42327677 37272648 50 PE
H3-ChIPseq-TRB3-ZF-Rep1_S1_L001 47498433 44531332 50 PE
H3-ChIPseq-TRB3-ZF-Rep2_S21_L003 34627333 29189300 50 PE
H3K27me3-ChIPseq-Col0-Rep1_S38_L004 35701023 24790314 50 PE
H3K27me3-ChIPseq-Col0-Rep2_S34_L004 41264013 29057697 50 PE
H3K27me3-ChIPseq-fwa-Rep1_S18_L001 12199070 11197483 50 PE
H3K27me3-ChIPseq-fwa-Rep1_S50_L003 23720150 19302200 50 PE
H3K27me3-ChIPseq-fwa-Rep2_S48_L003 24850552 19542262 50 PE
H3K27me3-ChIPseq-TRB1-ZF-Rep1_S23_L001 10667079 9917509 50 PE
H3K27me3-ChIPseq-TRB1-ZF-Rep1_S51_L003 23457045 18554823 50 PE
H3K27me3-ChIPseq-TRB1-ZF-Rep2_S53_L003 23019570 16957584 50 PE
H3K27me3-ChIPseq-trb123-Rep1_S37_L004 38883467 22939207 50 PE
H3K27me3-ChIPseq-trb123-Rep2_S35_L004 41241913 24042944 50 PE
H3K27me3-ChIPseq-TRB2-ZF-Rep1_S21_L001 10871814 9971853 50 PE
H3K27me3-ChIPseq-TRB2-ZF-Rep1_S71_L003 19099123 15283203 50 PE
H3K27me3-ChIPseq-TRB2-ZF-Rep2_S75_L003 18850368 12894861 50 PE
H3K27me3-ChIPseq-TRB3-ZF-Rep1_S22_L001 10819520 9975556 50 PE
H3K27me3-ChIPseq-TRB3-ZF-Rep1_S69_L003 19603897 15788879 50 PE
H3K27me3-ChIPseq-TRB3-ZF-Rep2_S89_L003 15442987 11743192 50 PE
H3K4me3-ChIPseq-Col0-Rep2_S20_L002 7074726 6173219 50 PE
H3K4me3-ChIPseq-Col0_S18_L002 30886642 27767146 50 PE
H3K4me3-ChIPseq-fwa-Rep1_S11_L001 17925369 16471918 50 PE
H3K4me3-ChIPseq-fwa-Rep1_S45_L003 26780511 22388582 50 PE
H3K4me3-ChIPseq-fwa-Rep2_S55_L003 22285816 17327546 50 PE
H3K4me3-ChIPseq-jmj14_S20_L002 27315172 23471069 50 PE
H3K4me3-ChIPseq-TRB1-ZF-Rep1_S17_L001 14752705 13237462 50 PE
H3K4me3-ChIPseq-TRB1-ZF-Rep1_S66_L003 20179690 16032270 50 PE
H3K4me3-ChIPseq-TRB1-ZF-Rep2_S84_L003 17038554 13335630 50 PE
H3K4me3-ChIPseq-trb123-Rep2_S19_L002 7676034 6723712 50 PE
H3K4me3-ChIPseq-trb123_S19_L002 27815135 24913917 50 PE
H3K4me3-ChIPseq-TRB2-ZF-Rep1_S13_L001 16000826 14861136 50 PE
H3K4me3-ChIPseq-TRB2-ZF-Rep1_S64_L003 20441881 16113650 50 PE
H3K4me3-ChIPseq-TRB2-ZF-Rep2_S82_L003 17538792 14787028 50 PE
H3K4me3-ChIPseq-TRB3-ZF-Rep1_S58_L003 22087670 18985369 50 PE
H3K4me3-ChIPseq-TRB3-ZF-Rep2_S81_L003 17580777 13033157 50 PE
H3K4me3-ChIPseq-TRB3-ZF_S14_L001 15987241 14814174 50 PE
FLAG ChIPseq Col0 Rep3 46771421 40100716 50 PE
FLAG ChIPseq Col0 Rep4 54934418 47809577 50 PE
FLAG ChIPseq TRB1 Rep 3 78667086 64173722 50 PE
FLAG ChIPseq TRB1 Rep 4 73527414 65109334 50 PE
FLAG ChIPseq TRB2 Rep 3 53241188 46236940 50 PE
FLAG ChIPseq TRB2 Rep 4 63153116 54914060 50 PE
FLAG ChIPseq TRB3 Rep 3 69019576 63199575 50 PE
FLAG ChIPseq TRB3 Rep 4 66253935 60388459 50 PE
Myc ChIPseq Col-0 93394114 33138911 50 PE
Myc ChIPseq JMJ14 55117705 30215610 50 PE
Myc ChIPseq JMJ14 in TRB1-ZF Rep1 43320387 22634743 50 PE
Myc ChIPseq JMJ14 in TRB1-ZF Rep2 76549695 46064156 50 PE
Myc ChIPseq JMJ14 in TRB2-ZF 41796529 22959777 50 PE
Myc ChIPseq JMJ14 in TRB3-ZF 28828824 13271033 50 PE
Input Col-0 84839846 66006607 50 PE
Input JMJ14 35905417 27954905 50 PE
Input JMJ14 in TRB1-ZF Rep1 67743003 51733393 50 PE
Input JMJ14 in TRB1-ZF Rep2 84076083 63987736 50 PE
Input JMJ14 in TRB2-ZF 74600658 54443896 50 PE
Input JMJ14 in TRB3-ZF 57503254 42177382 50 PE

Antibodies

Anti-H3K27me3 (Millipore Sigma)
Anti-H3 (Abcam)

	Anti-H3K4me3 (Millipore Sigma)<br>anti-FLAG M2 (Sigma)<br>Anti-Myc (Cell Signaling)
Peak calling parameters	MACS2: '-f BAM -g 1.3e+8 -q 0.05 --extsize 147'
Data quality	All identified peaks in the study were called with a qval threshold of 0.01 (FDR 1%).
Software	Bowtie (v1.1.2),<br>Samtools (v1.9)<br>MACS2 (v2.1.1)<br>ChIPseeker<br>deeptools (v2.5.1).<br>bedtools (v2.26.0)<br>MaxQuant
